# Dynamic quantitative photoacoustic tomography under sparse sampling via residual-driven Kalman filter assisted reconstruction

**DOI:** 10.1016/j.pacs.2026.100893

**Published:** 2026-10-03

**Authors:** Ye Wu, Wenxing Bai, Pengfei Zhang, Limin Zhang, Dongyuan Liu, Feng Gao

**Affiliations:** a School of Precision Instrument and Optoelectronics Engineering, Tianjin University, Tianjin, 300072, China; b State Key Laboratory of Precision Measurement Technology and Instruments, Tianjin University, Tianjin, 300072, China; c School of Artificial Intelligence, Tianjin University, Tianjin, 300072, China

**Keywords:** Quantitative photoacoustic tomography, Dynamic imaging, Sparse-pattern sampling, Residual-driven adaptive Kalman filtering, PDE-constrained reconstruction, Single-element detection

## Abstract

Mechanically scanned photoacoustic tomography (PAT) provides high detection sensitivity and flexible system configurations, but its application to dynamic quantitative imaging is limited by the trade-off between temporal resolution and spatial sampling density. We propose a coordinated acquisition–estimation–reconstruction (AER) framework for dynamic quantitative PAT under sparse-pattern sampling. Sparse-pattern cyclic (SPC) acquisition collects baseline-referenced differential signals, while residual-driven adaptive Kalman filtering (RDAKF) adapts location-specific state-transition matrices at sampled locations and uses neighborhood-weighted transition information to propagate states at unsampled locations. The recovered spatially complete differential PA waveforms are then used to reconstruct absorption-coefficient perturbations through adjoint-based, PDE-constrained optimization. The framework was evaluated in numerical simulations, dynamic phantom experiments, and ex vivo tissue experiments using a single-element transmission-mode PAT system. Across multiple sparse-pattern configurations in the dynamic phantom experiment, structural similarity index values of 0.81–0.84 and root-mean-square errors of 0.017–0.023 mm⁻¹ were obtained. In the single-location-per-pattern configuration, the mean sparse-pattern acquisition rate was approximately 0.84 Hz. The framework tracked gradual, abrupt, periodic, and motion-associated variations, supporting the feasibility of dynamic quantitative PAT under measurement-limited conditions.

## 1 Introduction

Photoacoustic tomography (PAT) combines optical absorption contrast with ultrasonic detection, enabling structural and functional imaging at depths that are generally inaccessible to purely optical techniques [[1], [2], [3]]. Through sensitivity to endogenous chromophores such as hemoglobin and melanin, PAT has been investigated for oxygen-metabolism assessment, hemodynamic monitoring, and tumor imaging [[4], [5], [6]].

Building on these capabilities, dynamic quantitative photoacoustic tomography (QPAT) aims to recover the temporal evolution of optical absorption properties and thereby characterize time-varying physiological or experimental processes [7,8]. Mechanically scanned systems, however, face a pronounced trade-off between temporal sampling rate and spatial measurement density. Multi-element arrays enable faster acquisition but entail greater hardware cost, system complexity, and calibration requirements [9]. Single-element scanning systems offer high receive sensitivity and comparatively simple instrumentation, but sequential acquisition intrinsically couples spatial sampling density to imaging speed: increasing the number of spatial measurements reduces the achievable temporal sampling rate [10,11].

Under dynamic conditions, sparse sampling can produce spatially incomplete measurements and temporal inconsistency among reconstructed frames, exacerbating the ill-posedness of the inverse problem and degrading quantitative accuracy [[12], [13], [14]]. A central challenge in dynamic QPAT is therefore to recover temporally consistent signals and stable quantitative images under severe undersampling.

Current approaches to dynamic photoacoustic imaging with sparse measurements include interpolation-based completion, spatiotemporal regularization, low-rank reconstruction, recursive state estimation, and deep learning. Interpolation is straightforward but relies on local spatial or temporal smoothness and may attenuate rapid or localized transients. Spatiotemporal regularization and low-rank methods exploit inter-frame correlations to stabilize sparse-data reconstruction [[15], [16], [17]], but they typically require batch optimization and may suppress weak dynamics when the imposed prior is overly restrictive. Kalman-type methods recursively combine model predictions with current observations and have been applied to photoacoustic denoising, state estimation, and dynamic-sequence filtering [18,19]. Their performance, however, depends strongly on prescribed state-transition and observation models, which may not represent spatially heterogeneous and nonstationary dynamics adequately [20,21]. Existing QPAT studies have largely focused on stabilizing optical-parameter inversion at individual states [22]. Deep-learning approaches provide strong representational capacity, but their use may be limited by dependence on training data, insufficient generalization, and limited physical interpretability [[23], [24], [25]].

To address the coupled challenges of sparse spatial sampling and spatially heterogeneous, nonstationary waveform dynamics, we propose an acquisition–estimation–reconstruction (AER) framework for dynamic QPAT. The framework integrates three sequentially coupled and functionally complementary stages. First, sparse-pattern cyclic (SPC) acquisition is implemented in a mechanically scanned, single-element, transmission-mode PAT system to collect differential PA signals. Each sparse pattern contains only a small subset of candidate locations, and the predefined sparse patterns are acquired cyclically in sequence, progressively providing direct measurements across the candidate field while maintaining a high pattern-level acquisition rate. Second, a residual-driven adaptive Kalman filter (RDAKF) recovers spatially complete differential PA waveforms by combining recursive state prediction, residual-driven adaptation of location-specific state-transition matrices at sampled locations, and neighborhood-weighted transition fusion to support state propagation at unsampled locations. As the sparse patterns cycle, direct residual-driven adaptation progressively reaches the entire candidate field. Finally, the recovered full-field differential waveforms are supplied to PDE-constrained dynamic QPAT, in which a physical forward model links absorption-coefficient perturbations to differential PA observations and adjoint-based optimization enables quantitative inversion. This coordinated workflow supports temporally consistent waveform recovery and spatially resolved quantitative reconstruction under severe undersampling, including the limiting configuration of S= 1, where each sparse pattern contains only one direct spatial measurement.

The framework was evaluated through numerical simulations, dynamic phantom experiments, and ex vivo tissue experiments. Across the tested sparse-pattern configurations, it provided stable dynamic tracking and model-consistent quantitative reconstruction, supporting further investigation of measurement-efficient dynamic PAT.

## 2 Methods

### 2.1 System configuration

Fig. 1(a) presents the mechanically scanned, single-element, transmission-mode two-dimensional (2D) photoacoustic imaging system. A Q-switched Nd:YAG laser operating at 1064 nm served as the excitation source, with a pulse duration of 7 ns and a repetition rate of 20 Hz (Dawa-200, Beamtech Optronics, China). Mirrors M1 and M2 directed the beam to a beam-expansion and diffusion (BE-DF) module, which expanded and homogenized the illumination over the phantom surface. The illumination spot was approximately 3 cm in diameter, and the incident surface fluence was approximately 7.69 mJ⋅cm^−2^ per pulse. PA signals were detected with a single-element unfocused ultrasonic transducer centered at 2 MHz (UG2P25S, ULSO Tech, China). The single-channel configuration avoids interelement sensitivity and timing variations while retaining the high receive sensitivity of single-element detection. The transducer was mounted on a three-axis motorized translation stage for precise positioning and 2D planar scanning. The commanded translation speed was 50 cm s^−1^, with acceleration and deceleration of 200 and 50 cm/s^2^, respectively. The lower deceleration was used to reduce mechanical vibration and positioning disturbances during stopping. The acquired PA signals were amplified by a preamplifier (ATA-5560, Aigtek, China) and digitized at 40 MS/s (USB8912, ART Technology, China). At each sampled location, ten PA waveforms generated by consecutive laser pulses were acquired over 0.5 s and averaged to form one measurement. Stage motion and waveform acquisition were performed sequentially: the stage remained stationary during acquisition and moved to the next location only after waveform averaging and data transfer. Translation time depended on the travel distance and prescribed stage velocity, as detailed in Supplementary Note S1 and Fig. S1.

**Fig. 1:**
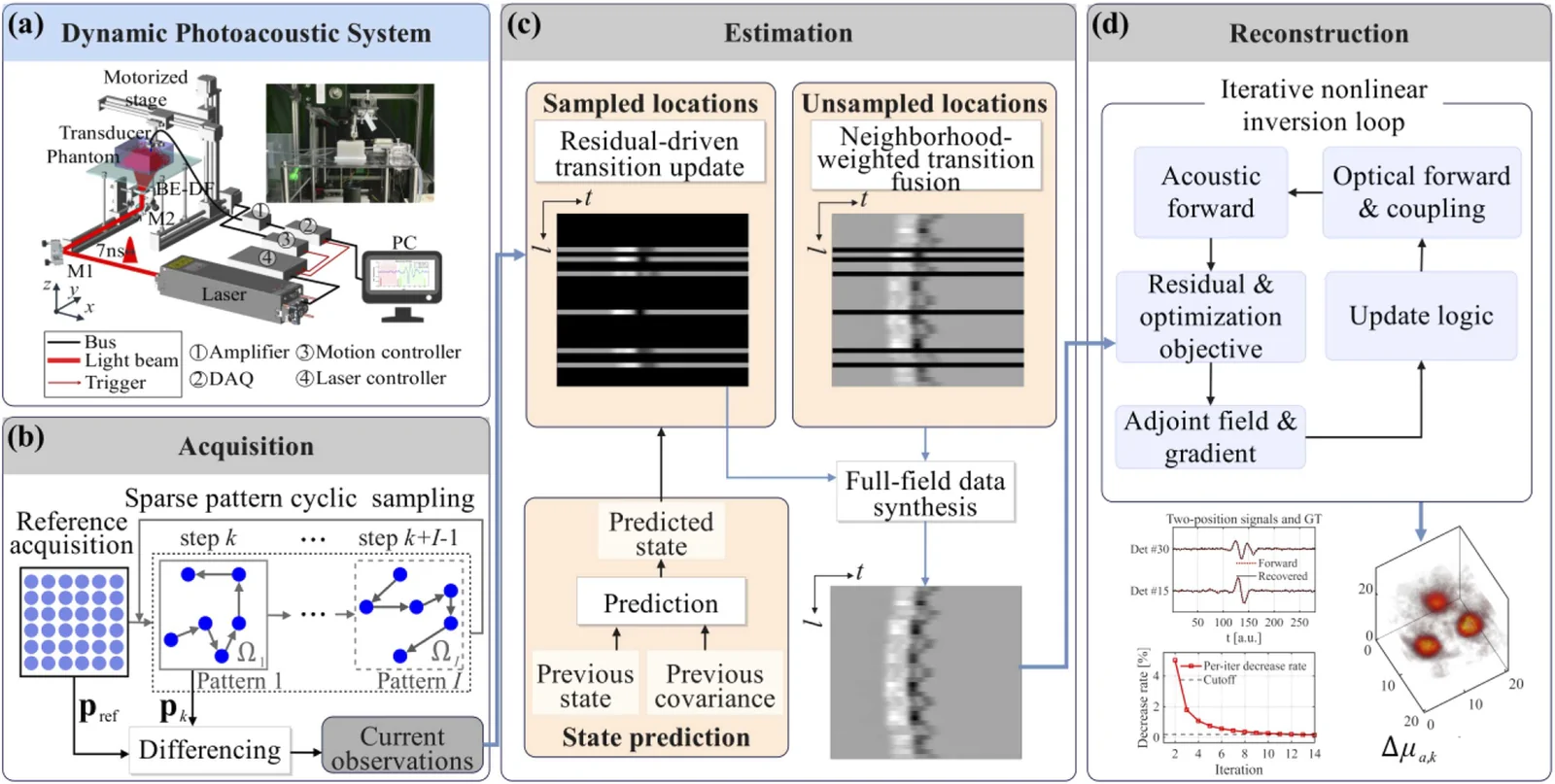
Overview of the acquisition–estimation–reconstruction (AER) framework and experimental photoacoustic (PA) imaging system. (a) Schematic of the mechanically scanned, single-element, transmission-mode PA tomography system; the inset shows the experimental setup. (b) Acquisition module: a full-field reference scan is followed by sparse-pattern cyclic (SPC) acquisition, in whichthe predefined sparse patterns are cycled through sequentially. The acquired waveforms are referenced to their baseline counterparts to obtain sparse differential PA observations. (c) Estimation module: residual-driven adaptive Kalman filtering (RDAKF) combines recursive state prediction, residual-driven adaptation of local transition matrices at sampled locations, and neighborhood-weighted transition fusion at unsampled locations. The resulting transition models propagate local states and produce a spatially complete differential PA waveform set after each sparse-pattern acquisition. (d) Reconstruction module: the recovered full-field waveforms are supplied to partial differential equation (PDE)-constrained dynamic quantitative photoacoustic tomography (QPAT). The iterative nonlinear inversion comprises optical forward modeling, optical–acoustic coupling, acoustic propagation, residual evaluation, adjoint-gradient calculation, and update of the absorption-coefficient perturbation, $\Delta \mu_{a,k}$. The lower panels show representative agreement between predicted and measured PA waveforms, residual convergence, and the reconstructed three-dimensional absorption-coefficient perturbation.

### 2.2 Data acquisition using sparse-pattern cyclic sampling

As illustrated in Fig. 1(b), the scanning plane contains L candidate spatial locations, denoted by $\Xi ={\left\{\xi_{l}\right\}}_{l=1}^{L}$, corresponding to the discrete positions visited by the single-element transducer. To promote progressive spatial coverage under sparse-sampling constraints, these locations are ordered into a global sequence using farthest-point sampling. At each selection step, the next location maximizes its minimum Euclidean distance from the previously selected set, thereby distributing the sampled positions across the measurement domain.

The ordered sequence is partitioned into sparse sampling patterns ${\left\{\Omega_{i}\right\}}_{i=1}^{I}$, each containing S locations, except possibly the final pattern when L is not exactly divisible by S. The patterns partition the candidate location set, satisfying $\Omega_{i}\cap \Omega_{j}=\emptyset$ for i≠j and ${⋃}_{i=1}^{I}\Omega_{i}=\Xi$. During dynamic acquisition, the transducer sequentially scans the locations in each predefined sparse pattern. The predefined pattern sequence is then repeated cyclically, and completion of one pattern defines one sparse acquisition step. Accordingly, each candidate location is directly measured once during one complete pass through all predefined sparse patterns. With one-based indexing, the pattern acquired at the k-th step is $\Omega_{i_{k}}$, where $i_{k}=\mathrm{mod}\left(k-1,I\right)+1$. At the k-th acquisition step, PA waveform measurements at the locations within pattern $\Omega_{i_{k}}$ are acquired sequentially, and the pattern-completion time $T_{k}$, corresponding to the completion time of the measurement at the final visited location, is assigned as the time coordinate of the corresponding estimated and reconstructed frame. For S>1, measurements acquired earlier within the same pattern therefore have finite temporal offsets relative to $T_{k}$.

At the k-th acquisition step, only locations $\xi_{l}\in \Omega_{i_{k}}$ are directly sampled. The PA waveform acquired at location $\xi_{l}$ is denoted by $p_{l,k}\in R^{M}$, where M is the number of data points along the acoustic time-of-flight axis. A full reference scan is acquired before the dynamic sequence, and the baseline waveform at location $\xi_{l}$ is denoted by $p_{l,\mathrm{ref}}$. The sparse differential observation is then defined as $∆p_{l,k}=p_{l,k}-p_{l,\mathrm{ref}}$ for $\xi_{l}\in \Omega_{i_{k}}$. After each sparse-pattern acquisition, RDAKF combines the new observations with recursively propagated states to estimate differential PA waveforms at all L candidate locations, producing a spatially complete waveform set for dynamic QPAT reconstruction.

The SPC acquisition rate is defined as the pattern-level sparse acquisition rate, rather than the full-field direct-measurement rate. The sparse acquisition rate is approximated as $f_{\mathrm{spc}}\approx 1/\left(T_{\mathrm{samp}}+T_{\mathrm{travel}}\right)$, where $T_{\mathrm{samp}}$ is the mean signal-acquisition time per pattern and $T_{\mathrm{travel}}$ is the mean mechanical-travel overhead. Together, these terms account for waveform acquisition at all locations in $\Omega_{i}$, point-to-point stage motion, and inter-pattern transition latency. Each candidate location is directly measured once during one complete pass through all predefined sparse patterns, and S controls the trade-off between direct spatial sampling density and pattern-level temporal resolution. In the limiting single-location-per-pattern configuration (S=1), each sparse pattern contains one directly observed location, providing the highest pattern-level acquisition rate but the most severe spatial undersampling.

### 2.3 Residual-driven adaptive Kalman filtering

After the k-th sparse-pattern acquisition step, RDAKF estimates the full-field differential photoacoustic (PA) waveforms using the measurements acquired in the current pattern, providing a spatially complete waveform dataset for PDE-constrained dynamic QPAT reconstruction at $T_{k}$. At sampled locations, pre-adaptation model residuals are used to update the local state-transition matrices; the priori waveform state and covariance are then recomputed with the updated matrix and corrected through the Kalman measurement update. At unsampled locations, each retained transition matrix is relaxed toward a distance-weighted estimate formed from valid neighboring transition matrices. The waveform state is propagated from the preceding acquisition step without measurement correction. The resulting states, transition matrices, and covariance matrices are propagated recursively to the next acquisition step, yielding a dynamic sequence of full-field differential PA waveforms.

Upon completion of the k-th sparse-pattern acquisition, RDAKF estimates the full-field differential PA waveform matrix $X_{k}$, expressed as

(1) $$X_{k}={\left[x_{1,k|k},x_{2,k|k},\ldots ,x_{L,k|k}\right]}^{T}\in R^{L\times M},$$

where $x_{l,k|k}\in R^{M}$ denotes the posterior differential PA waveform-state estimate at candidate location $\xi_{l}$, and M is the number of data points in each PA waveform.

For each candidate location, RDAKF maintains a location-specific transition matrix $A_{l,k}\in R^{M\times M}$ that describes the temporal evolution of the differential waveform state between successive acquisition steps. At initialization, all transition matrices $A_{l,0}$ are assigned the same scaled identity matrix as

(2) $$A_{l,0}=a_{0}I_{M},l=1,\ldots ,L,$$

where $a_{0}$ is the transition-initialization coefficient and $I_{M}$ is the M×M identity matrix. In the absence of prior information on waveform dynamics, $a_{0}$ is set to 1, corresponding to an identity transition model with no initial amplification or attenuation.

The location-specific state-transition matrix $A_{l,k-1}$ retained after the (k−1)-th acquisition step is used to predict the waveform state at step k, yielding the provisional a priori estimate ${\tilde{x}}_{l,k|k-1}$,

(3) $${\tilde{x}}_{l,k|k-1}=A_{l,k-1}x_{l,k-1|k-1},$$

where $x_{l,k-1|k-1}\in R^{M}$ is the posterior waveform-state estimate retained after the (k−1)-th acquisition step, and ${\tilde{x}}_{l,k|k-1}\in R^{M}$ is the provisional a priori estimate for step k predicted from the preceding posterior state.

The corresponding provisional a priori state-error covariance ${\tilde{P}}_{l,\mathrm{k|k}-1}$ is propagated as

(4) $${\tilde{P}}_{l,k|k-1}=A_{l,k-1}P_{l,k-1|k-1}A_{l,k-1}^{T}+Q,$$

where ${\tilde{P}}_{l,\mathrm{k|k}-1}\in R^{M\times M}$ is the provisional a priori state-error covariance for step k, conditioned on information available through step k−1 at candidate location $\xi_{l}$; $P_{l,k-1\mathrm{|k}-1}\in R^{M\times M}$ is the posterior state-error covariance retained after the (k−1)-th acquisition step; $A_{l,k-1}^{T}$ denotes the transpose of $A_{l,k-1}$; and $Q\in R^{M\times M}$ is the process-noise covariance accounting for model uncertainty and unmodeled waveform variations [21,26]. At initialization, the posterior covariance and process-noise covariance are set to the scaled identity matrices $P_{l,0|0}=P_{0}I_{M}$ and $Q=Q_{0}I_{M}$**,** respectively.

For each sampled location $\xi_{l}\in \Omega_{i_{k}}$, the local state-transition matrix is adapted before the Kalman measurement update. The pre-adaptation model residual $e_{l,k}$ is defined as

(5) $$e_{l,k}=y_{l,k}-H{\tilde{x}}_{l,\mathrm{k|k}-1},$$

where $y_{l,k}$ is the differential PA waveform observed at sampled location $\xi_{l}$ during the k-th acquisition step. In the reported implementation, the observation matrix is fixed as $H=I_{M}$, so adaptation is confined to the state-transition dynamics.

Before Kalman measurement correction, the pre-adaptation residual is used to update the local state-transition matrix. At the k-th acquisition step, the regularized prediction-error objective $J_{l,k}\left(A_{l,k-1}\right)$**,** evaluated at the transition matrix retained from the preceding step, is defined as

(6) $$J_{l,k}\left(A_{l,k-1}\right)=\frac{1}{2}{∥y_{l,k}-HA_{l,k-1}x_{l,k-1|k-1}∥}_{2}^{2}+\frac{\lambda }{2}{∥A_{l,k-1}∥}_{F}^{2}.$$

The gradient of this objective with respect to the retained transition matrix $A_{l,k-1}$ is

(7) $$\nabla_{A_{l,k-1}}J_{l,k}=-H^{T}\left(y_{l,k}-HA_{l,k-1}x_{l,k-1|k-1}\right)x_{l,k-1|k-1}^{T}+\lambda A_{l,k-1}.$$

Here, $\|\cdot {\|}_{2}$ denotes the Euclidean norm, $\|\cdot {\|}_{F}$ denotes the Frobenius norm, and $\nabla_{A_{l,k-1}}$ is the gradient operator with respect to $A_{l,k-1}$. The coefficient λ controls the Frobenius-norm penalty, limiting excessive growth of the state-transition matrix and reducing sensitivity to instantaneous residual fluctuations. In this study, λ was set to $1\times 10^{-3}$.

To scale the transition update according to the current prediction mismatch, the normalized residual magnitude $r_{l,k}$ is defined as

(8) $$r_{l,k}=\frac{\|e_{l,k}{\|}_{2}}{\|y_{l,k}{\|}_{2}+\epsilon }.$$

The corresponding residual-dependent learning rate $\eta_{l,k}$ is

(9) $$\eta_{l,k}=\eta_{0}\frac{r_{l,k}}{c_{\mathrm{scale}}+r_{l,k}+\epsilon }，$$

where $\eta_{0}$ is the base learning rate, $c_{\mathrm{scale}}$ is a dimensionless residual-saturation parameter controlling how rapidly $\eta_{l,k}$ approaches $\eta_{0}$ as $r_{l,k}$ increases, and ϵ is a small positive numerical floor used to prevent unstable normalization when a denominator approaches zero. In this study, $\eta_{0}=$ 0.06, $c_{\mathrm{scale}}=$ 0.8, and ϵ= 10^−6^.

Using recursive prediction-error identification and normalized stochastic-gradient adaptation, the current-step transition matrix $A_{l,k}$ is updated through one normalized gradient step as

(10) $$A_{l,k}=A_{l,k-1}-\eta_{l,k}\frac{\nabla_{A_{l,k-1}}J_{l,k}}{{∥\nabla_{A_{l,k-1}}J_{l,k}∥}_{F}+\epsilon }.$$

After adaptation, the updated matrix $A_{l,k}$ is retained as the location-specific transition matrix for the k-th acquisition step and is used to recompute the a priori waveform-state estimate $x_{l,\mathrm{k|k}-1}$ and state-error covariance $P_{l,\mathrm{k|k}-1}$ as

(11) $$x_{l,k|k-1}=A_{l,k}x_{l,k-1|k-1},$$

(12) $$P_{l,k|k-1}=A_{l,k}P_{l,k-1|k-1}A_{l,k}^{T}+Q.$$

The provisional a priori state estimate in Eqs. (3), computed using the transition matrix $A_{l,k-1}$ retained from the preceding acquisition step, is used to evaluate the pre-adaptation residual and update the local transition matrix. The updated matrix $A_{l,k}$ is then used to recompute the a priori state and covariance in Eqs. (11) and (12) for the subsequent Kalman measurement correction. Based on the recomputed a priori waveform-state estimate $x_{l,\mathrm{k|k}-1}$, the Kalman innovation $\nu_{l,k}$, defined as the difference between the current observation and its a priori prediction, is given by

(13) $$\nu_{l,k}=y_{l,k}-{\mathrm{Hx}}_{l,k|k-1}.$$

The corresponding innovation covariance and Kalman gain are then calculated as

(14) $$S_{l,k}={\mathrm{HP}}_{l,\mathrm{k|k}-1}H^{T}+R,$$

(15) $$C_{l,k}=P_{l,\mathrm{k|k}-1}H^{T}S_{l,k}^{-1},$$

where $R\in R^{M\times M}$ is the measurement-noise covariance matrix, $H^{T}$ is the transpose of the observation matrix H, $S_{l,k}\in R^{M\times M}$ is the innovation covariance**,** and $C_{l,k}\in R^{M\times M}$ is the Kalman gain at location $\xi_{l}$ and the k-th acquisition step. The same measurement-noise covariance is used at all candidate locations and is specified as $R=R_{0}I_{M}$, where $R_{0}$> 0 is the prescribed covariance scale.

At the k-th acquisition step, a posteriori waveform-state estimate $x_{l,\mathrm{k|k}}$ and its state-error covariance $P_{l,\mathrm{k|k}}$ at the sampled location are obtained through the Kalman measurement update as

(16) $$x_{l,k|k}=x_{l,k|k-1}+C_{l,k}\nu_{l,k},$$

(17) $$P_{l,\mathrm{k|k}}=\left(I_{M}-C_{l,k}H\right)P_{l,\mathrm{k|k}-1}.$$

Thus, the pre-adaptation residual $e_{l,k}$ is used for local state-transition-matrix adaptation, whereas the Kalman innovation $\nu_{l,k}$, computed from the a priori estimate obtained with the updated matrix $A_{l,k}$, is used for measurement correction.

For an unsampled location $\xi_{l}\notin \Omega_{i_{k}}$, neither residual-driven transition adaptation nor measurement correction is performed. Instead, the waveform state and state-error covariance are propagated using spatially shared transition information. A fixed neighborhood graph is constructed from the physical coordinates of the candidate scanning locations. At the k-th acquisition step, the active neighbor set $N_{l,k}$ includes only neighboring locations that have received at least one valid direct observation through the current step. For each $j\in N_{l,k}$, the distance-dependent weight $w_{\mathrm{lj},k}$ is defined as

(18) $$w_{\mathrm{lj},k}=\frac{\exp\left[-\|\xi_{l}-\xi_{j}{\|}_{2}^{2}/\left(2\sigma_{\mathrm{space}}^{2}\right)\right]}{\sum_{q\in {\mathbb{N}}_{l,k}}\exp\left[-\|\xi_{l}-\xi_{q}{\|}_{2}^{2}/\left(2\sigma_{\mathrm{space}}^{2}\right)\right]},\;j\in {\mathbb{N}}_{l,k},$$

where $\xi_{l}$ and $\xi_{j}$ are the physical coordinates of the unsampled location and a valid neighboring location, respectively, and ${∥\xi_{l}-\xi_{j}∥}_{2}$ is their Euclidean distance on the scanning plane. The parameter $\sigma_{\mathrm{space}}$ controls the spatial decay of the weights, assigning larger weights to closer neighbors. Here, q is a dummy summation index over the valid neighbors in $N_{l,k}$, and the normalized weights satisfy $\sum_{j\in N_{l,k}}w_{l,j,k}=1$. The distance-weighted transition matrix is then calculated as

(19) $$A_{l,k}^{\mathrm{nbr}}=\sum_{j\in {\mathbb{N}}_{l,k}}w_{\mathrm{lj},k}A_{j,k}.$$

The transition matrix $A_{l,k}$ at the unsampled location is relaxed toward the distance-weighted neighborhood estimate as

(20) $$A_{l,k}=\left(1-\gamma_{A}\right)A_{l,k-1}+\gamma_{A}A_{l,k}^{\mathrm{nbr}},\;{0\leq \gamma }_{A}\leq 1,$$

where $\gamma_{A}$ is the fusion weight controlling the relative contributions of the locally retained transition matrix and the neighborhood-weighted estimate.

At the k-th acquisition step, the waveform state $x_{l,\mathrm{k|k}-1}$ at the unsampled location is predicted using the fused transition matrix, and the associated state-error covariance $P_{l,\mathrm{k|k}-1}$ is propagated as

(21) $$x_{l,k|k-1}=A_{l,k}x_{l,k-1|k-1},$$

(22) $$P_{l,k|k-1}=A_{l,k}P_{l,k-1|k-1}A_{l,k}^{T}+Q.$$

At an unsampled location, no current observation is available; therefore, measurement correction is skipped. The propagated a priori quantities $x_{l,k|k-1}$ and $P_{l,\mathrm{k|k}-1}$ are assigned directly to the posterior quantities $x_{l,k|k}$ and $P_{l,\mathrm{k|k}}$ at the k-th step as

(23) $$x_{l,k|k}=x_{l,k|k-1},\;P_{l,k|k}=P_{l,k|k-1}.$$

After each sparse-pattern acquisition, the posterior states are assembled into the full-field waveform matrix $X_{k}$ as

(24) $$X_{k}={\left[x_{1,k|k},x_{2,k|k},\ldots ,x_{L,k|k}\right]}^{T}\in {\mathbb{R}}^{L\times M}.$$

This assembly produces a spatially complete differential PA waveform matrix for subsequent PDE-constrained dynamic QPAT reconstruction. The location-specific transition and state-error covariance matrices are retained in full M×M form, allowing cross-sample dependencies to be represented during state prediction and the corresponding uncertainty correlations to be preserved during measurement correction.

### 2.4 Adjoint-based nonlinear differential reconstruction

The absorption-coefficient perturbation $\Delta \mu_{a,k}\in R^{N}$ is represented by nodal values on the finite-element mesh at the k-th acquisition step, where N is the number of mesh nodes. It is reconstructed by solving a PDE-constrained nonlinear inverse problem using the spatially complete differential PA waveforms estimated by RDAKF as observations. The nonlinear forward operator $F\left(\Delta \mu_{a,k}\right)$ maps the absorption perturbation to predicted differential PA waveforms through optical-fluence calculation, differential initial-pressure generation, and acoustic propagation. At each acquisition step, the perturbation is reconstructed by minimizing the following data-misfit objective function:

(25) $$L\left(\Delta \mu_{a,k}\right)=\frac{1}{2}\|F\left(\Delta \mu_{a,k}\right)-∆p_{k}{\|}_{2}^{2},$$

where $∆p_{k}$ denotes the vectorized recovered full-field differential PA waveform data obtained after the k-th sparse-pattern acquisition at completion time $T_{k}$.

For a given absorption-coefficient perturbation $\Delta \mu_{a,k}$, the total absorption-coefficient distribution is $\mu_{a,k}=\mu_{a,\mathrm{ref}}+\Delta \mu_{a,k}$, where $\mu_{a,\mathrm{ref}}\in R^{N}$ is the reference distribution. The optical fluence $\Phi_{k}\in R^{N}$ is obtained by solving the finite-element system $K(\mu_{a,k})\Phi_{k}=q$ subject to a Robin boundary condition, where $K(\mu_{a,k}){\in R}^{N\times N}$ is the optical stiffness matrix assembled from the current absorption-coefficient distribution and $q\in R^{N}$ is the source vector. The differential initial-pressure distribution relative to the reference state is $\delta p_{\mathrm{init},k}=\Gamma \left(\mu_{a,k}⊙\Phi_{k}-\mu_{a,\mathrm{ref}}⊙\Phi_{\mathrm{ref}}\right)$, where $\Gamma {\in R}^{N}$ is the nodal Grüneisen-parameter distribution, $\Phi_{\mathrm{ref}}\in R^{N}$ is the reference fluence, and ⊙ denotes element-wise multiplication. The predicted differential PA waveforms are given by $F\left(\Delta \mu_{a,k}\right)=H\delta p_{\mathrm{init},k}$, where H is the acoustic forward operator that maps differential initial pressure to the detected waveforms.

The minimization problem is solved using an iterative adjoint-state method [[27], [28], [29]]. At reconstruction iteration l for the k-th acquisition step, the data residual is defined as $d_{k}^{\left(l\right)}=F\left(\Delta \mu_{a,k}^{\left(l\right)}\right)-∆p_{k}$. The acoustic adjoint field $p_{k}^{*,(l)}$ is computed by applying the transpose of the discrete acoustic forward operator to the data-misfit vector as

(26) $$p_{k}^{*,(l)}=H^{T}d_{k}^{\left(l\right)},$$

where $H^{T}$ denotes the transpose of the discrete acoustic forward operator, and the superscript * identifies an adjoint variable.

The reference-state contribution is fixed throughout reconstruction and therefore has zero derivative with respect to $\Delta \mu_{a,k}$. At the l-th iteration, the optical adjoint field $\Phi_{k}^{*,(l)}{\in R}^{N}$ is obtained by solving the transposed finite-element optical system as

(27) $$K{\left(\mu_{a,k}^{\left(l\right)}\right)}^{T}\Phi_{k}^{*,(l)}=-\Gamma ⊙\mu_{a,k}^{\left(l\right)}⊙p_{k}^{*,(l)}.$$

The objective-function gradient with respect to the absorption-coefficient perturbation is then evaluated as

(28) $$\nabla_{\Delta \mu_{a,k}}L^{\left(l\right)}=\Gamma ⊙\Phi_{k}^{\left(l\right)}⊙p_{k}^{*,(l)}+\Phi_{k}^{\left(l\right)}⊙\Phi_{k}^{*,(l)}.$$

The absorption-coefficient perturbation $\Delta \mu_{a,k}^{\left(l+1\right)}$ is updated by gradient descent as

(29) $$\Delta \mu_{a,k}^{\left(l+1\right)}=\Delta \mu_{a,k}^{\left(l\right)}-\sigma \nabla_{\Delta \mu_{a,k}}L^{\left(l\right)},$$

where σ is the relaxation step size. At each iteration, the total absorption distribution is updated, after which the optical forward field, predicted differential PA waveforms, acoustic adjoint variable, and optical adjoint field are recomputed. A node-wise nonnegativity projection is applied after each update because all absorption perturbations considered in this study are nonnegative relative to their reference states.

## 3 Results

### 3.1 Numerical simulation

To evaluate the AER framework numerically, a three-dimensional phantom containing three dynamically varying cylindrical absorbers was constructed (Fig. 2(a)). The phantom dimensions were 20 × 20 × 30 ${\mathrm{mm}}^{3}$. The absorbers had diameters of 2 mm and were centered at (5,12,11), (15,12,11), and (10,7,18) mm, respectively. Their absorption coefficients followed distinct temporal profiles (Fig. 2(b)), generating spatially separated PA responses with different dynamics. The background absorption and reduced scattering coefficients were $\mu_{a,\mathrm{bg}}=$0.005 ${\mathrm{mm}}^{-1}$ and $\;\mu_{s,\mathrm{bg}}^{\prime }=$1.0 ${\mathrm{mm}}^{-1}$, respectively. Optical fluence was calculated using a finite-element solution of the diffusion equation with a Robin boundary condition on a mesh containing N=105895 nodes. The resulting initial-pressure distributions were propagated acoustically using k-Wave on a 40 × 40 × 60 computational grid. The sound speed, mass density, and nodal Grüneisen-parameter vector were c=1500 $m∙s^{-1}$**,** ρ=1000 $\mathrm{kg}∙m^{-3}$, and $\Gamma ={\left[\Gamma_{1},\Gamma_{2},\ldots ,\Gamma_{N}\right]}^{T}\in R^{N}$, respectively, with $\Gamma_{i}=$0.0375 for all i=1,…,N. A finite-bandwidth detector response with a center frequency of 2 MHz and a fractional bandwidth of 80% was included. Frequency-dependent attenuation was modeled as $\alpha \left(f\right)=\alpha_{0}{\left(f/1\mathrm{MHz}\right)}^{\beta }$, with $\alpha_{0}=$0.75 dB/cm at 1 MHz and β=1.5. PA waveforms were simulated at 36 detection locations separated by d=3 mm center-to-center.

**Fig. 2:**
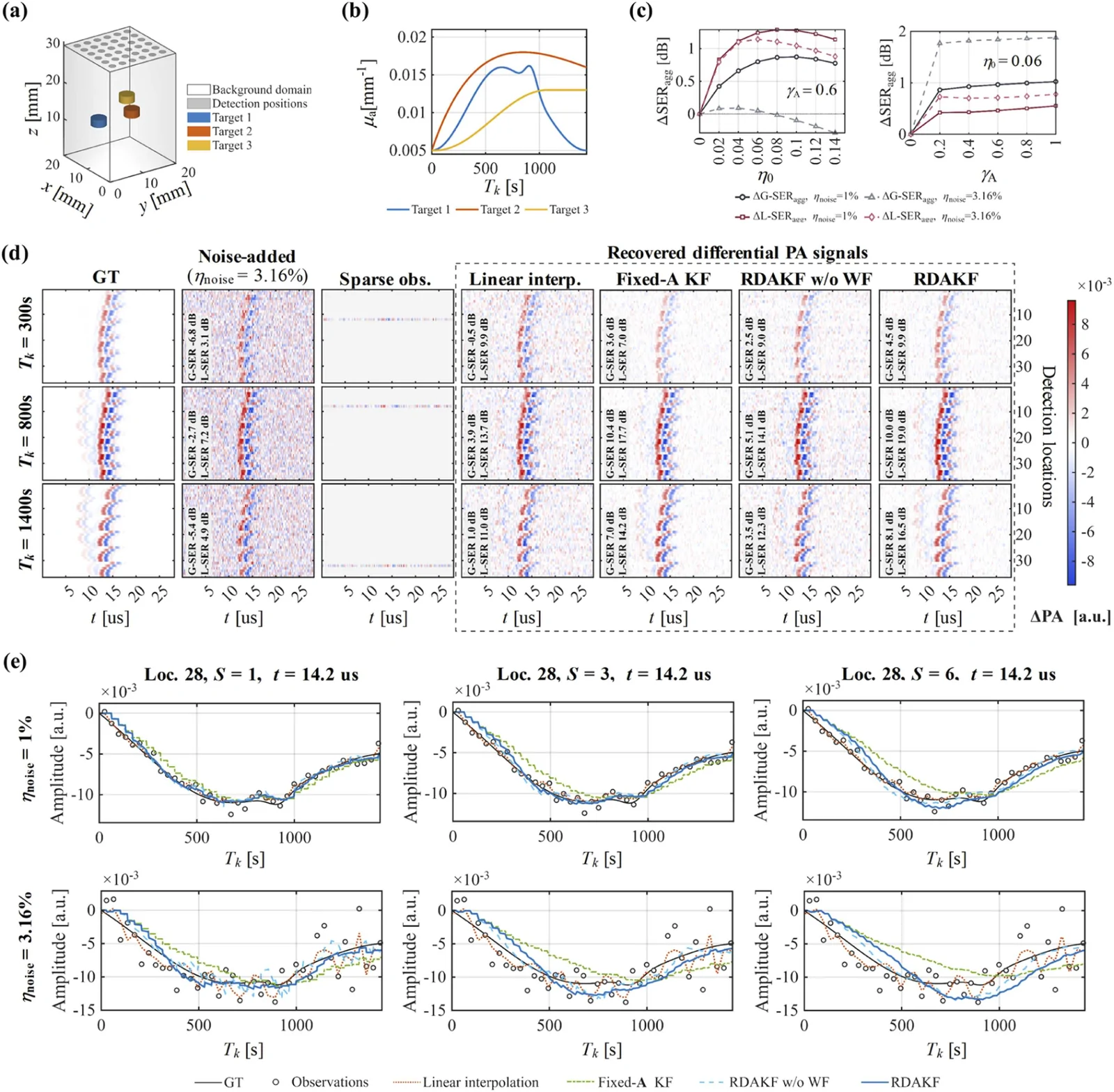
Simulation configuration and evaluation of full-field differential photoacoustic (PA) waveform recovery. (a) Three-dimensional simulation domain containing three absorbing targets and 36 candidate detection locations. (b) Prescribed temporal profiles of the absorption-coefficient perturbations for the three targets. (c) Effects of the base learning rate *η*_0_ and neighborhood-transition-fusion weight *γ*_A_ on residual-driven adaptive Kalman filter (RDAKF) performance, quantified using the aggregated global signal-to-error ratio improvement (ΔG-SER_agg_) and aggregated local signal-to-error ratio improvement(ΔL-SER_agg_) at *η*_noise_ = 1% and 3.16%. Each point represents the aggregated result over five independent simulation realizations. (d) Ground-truth (GT), noise-contaminated, sparsely observed, and recovered differential PA waveform maps at $T_{k}=300,\;800$, and 1400s for *η*_noise_ = 3.16%. The recovery methods include linear interpolation along the sparse-pattern acquisition-time dimension, Kalman filtering with a fixed state-transition matrix (fixed-**A** KF), RDAKF without neighborhood-weighted transition fusion (RDAKF w/o WF), and the full RDAKF configuration. The corresponding G-SER and L-SER values are indicated in each recovered map. (e) Temporal amplitude profiles at detection location 28 and a waveform time of *t* = 14.2 μs for *S* = 1, 3, and 6 at both noise levels. The curves compare the GT, sparse noisy observations, linear interpolation, fixed-A KF, RDAKF w/o WF, and the full RDAKF. For conditions evaluated over five independent noise realizations, the curves show the mean profiles, and the shaded bands indicate ±1 standard deviation.

The SPC strategy described in Section 2.2 was reproduced in simulation, with each location-level acquisition, including signal acquisition and mechanical translation, assigned a duration of ∆t=1 s. A full-field reference dataset $p_{0}^{\mathrm{GT}}$ was first acquired at all *L* detection locations. During the dynamic sequence, locations were visited cyclically using predefined patterns containing S= 1,3 or 6 locations. Zero-mean Gaussian noise was added to the simulated PA waveforms as

(30) $$p_{k}^{\mathrm{noisy}}=p_{k}^{\mathrm{GT}}+\sigma_{\mathrm{noise}}z_{k},\;{\left[z_{k}\right]}_{l,n}\sim N\left(0,1\right)，$$

where $p_{k}^{\mathrm{GT}}$ is the noise-free full-field PA waveform matrix at $T_{k}$, and superscript GT denotes ground truth. The noise matrix $z_{k}$ has the same dimensions and contains independent standard Gaussian entries. The noise standard deviation is $\sigma_{\mathrm{noise}}=\eta_{\mathrm{noise}}\mathrm{RMS}\left(p^{\mathrm{GT}}\right)$, where $\eta_{\mathrm{noise}}$ is the prescribed relative root-mean-square (RMS) noise level. The RMS value was calculated over all raw ground-truth PA data, including the reference frame. At each sampled location, the noisy reference waveform was subtracted from the noisy dynamic waveform to form the sparse differential observation.

RDAKF was used to recover the spatially complete differential PA waveform matrix from the sparse measurements. Three alternative signal-recovery methods were implemented for comparison: temporal linear interpolation along the sparse-pattern acquisition-time axis, a Kalman filter with a fixed state-transition matrix (fixed-A KF), and RDAKF without neighborhood-weighted transition fusion (RDAKF w/o WF).

For acquisition step k**,** the ground-truth (GT) differential PA signal matrix at sparse-pattern completion time $T_{k}$ is defined as $\Delta p_{k}^{\mathrm{GT}}=p_{k}^{\mathrm{GT}}-p_{0}^{\mathrm{GT}}.$ Full-field and target-focused recovery accuracy were quantified by the global and local signal-to-error ratios, respectively:

(31) $${G-\mathrm{SER}}_{k}=10\log_{10}\left[\frac{{∥\Delta p_{k}^{\mathrm{GT}}∥}_{F}^{2}}{{∥\;\Delta p_{k}-\Delta p_{k}^{\mathrm{GT}}∥}_{F}^{2}+\epsilon }\right],$$

(32) $${L-\mathrm{SER}}_{k}=10\log_{10}\left[\frac{{∥M_{k}⊙\Delta p_{k}^{\mathrm{GT}}∥}_{F}^{2}}{{∥M_{k}⊙\left(\;\Delta p_{k}-\Delta p_{k}^{\mathrm{GT}}\right)∥}_{F}^{2}+\epsilon }\right],$$

where $\Delta p_{k}$ denotes the recovered differential PA waveform matrix, ⊙ denotes element-wise multiplication, and ϵ is a small positive constant. G-SER assesses the complete detection-location–waveform map, whereas L-SER emphasizes the dynamically responsive target region. The local target-response mask was derived from the ground truth at each sparse-pattern step as

(33) $$M_{k}\left(l,m\right)=\left\{\begin{array}{cc} 1, & \left|\Delta p_{k}^{\mathrm{GT}}\left(l,m\right)\right|\geq 0.20\underset{l,m}{\max}\left|\Delta p_{k}^{\mathrm{GT}}\left(l,m\right)\right|,\\ 0, & \text{otherwise}. \end{array}\right)$$

The effects of base learning rate $\eta_{0}$ and fusion weight $\gamma_{A}$ were examined in Fig. 2(c) by varying one parameter while holding the other at its reference value. A joint analysis of $\eta_{0}$ and $\gamma_{A}$, including two-dimensional performance maps over the full tested ranges, is provided in Supplementary Note S2.8 and Fig. S6. For each noise realization, signal and error energies were first accumulated over the evaluated acquisition steps; the resulting energies were then pooled across realizations before conversion to decibels:

(34) $${SER}_{agg}=10\log_{10}\left(\frac{\sum_{r,k}E_{r,k}^{sig}}{\sum_{r,k}E_{r,k}^{err}+\epsilon }\right)$$

(35) $$\Delta {\mathrm{SER}}_{\mathrm{agg}}\left(x\right)={\mathrm{SER}}_{\mathrm{agg}}\left(x\right)-{\mathrm{SER}}_{\mathrm{agg}}\left(0\right)，$$

where $E_{r,k}^{sig}$ and $E_{r,k}^{err}$ denote the signal and error energies, respectively, at the k-th evaluated acquisition step of the r-th independent realization. Here, $\eta_{0}=$ 0 corresponds to Kalman filtering with a fixed transition matrix, whereas $\gamma_{A}=$ 0 disables neighborhood-weighted transition fusion. Increasing $\eta_{0}$ improved both ΔG-SER_agg_ and ΔL-SER_agg_, with a larger gain in ΔL-SER_agg_, indicating that residual-driven transition adaptation improved tracking of location-specific dynamics. At larger $\eta_{0}$, the gains saturated and decreased slightly under some conditions. With neighborhood radius fixed at 1.75d, increasing $\gamma_{A}$ strengthened transition sharing and improved both metrics, although the benefit plateaued at larger values. Thus, neighborhood transition information improved waveform prediction at unsampled locations, whereas further increases in fusion strength provided limited additional benefit. Based on Fig. 2(c) and the joint heatmaps in Supplementary Note S2.8, $\eta_{0}=$ 0.06 and $\gamma_{A}=$ 0.6 were selected from a broad high-performance region. Initialization and validation of P, Q, and R are detailed in Supplementary Note S2.7. The retained settings are summarized in Table S3, and covariance sensitivity results are shown in Figs. S2–S5.

For method comparison, the sampling condition was fixed at S= 1, corresponding to one directly sampled location per acquisition step, and the higher noise level $\eta_{\mathrm{noise}}=$ 3.16% was used for the representative waveform maps in Fig. 2(d). All methods recovered the principal waveform structure, but linear interpolation and RDAKF w/o WF showed larger deviations. Linear interpolation estimated missing samples from adjacent noisy observations and was therefore susceptible to noise propagation, amplitude smoothing, and temporal error. The fixed-A KF improved waveform stability and noise suppression, but its fixed transition model could not fully represent evolving dynamics. Full RDAKF combined residual-driven transition adaptation at sampled locations with neighborhood-weighted transition fusion at unsampled locations, providing a more favorable balance among waveform continuity, amplitude preservation, and dynamic tracking.

The quantitative results were consistent with these observations. At $T_{k}\;=$ 800 s, linear interpolation, fixed-**A** KF, RDAKF w/o WF, and full RDAKF achieved G-SER/L-SER values of 3.9/13.7, 10.4/17.7, 4.1/14.1, and 10.0/19.0 dB, respectively. At $T_{k}\;=$ 1400 s, the corresponding values were 1.0/11.0, 7.0/14.2, 3.5/12.3, and 8.1/16.5 dB. Although fixed-**A** KF yielded a marginally higher G-SER at $T_{k}\;=$ 800 s, full RDAKF achieved the highest L-SER at that time point and the highest G-SER and L-SER at $T_{k}\;=$ 1400 s. Together with the complete temporal evaluation in Fig. 3, these results show that full RDAKF provided the most consistent recovery across the global and local metrics.

**Fig. 3:**
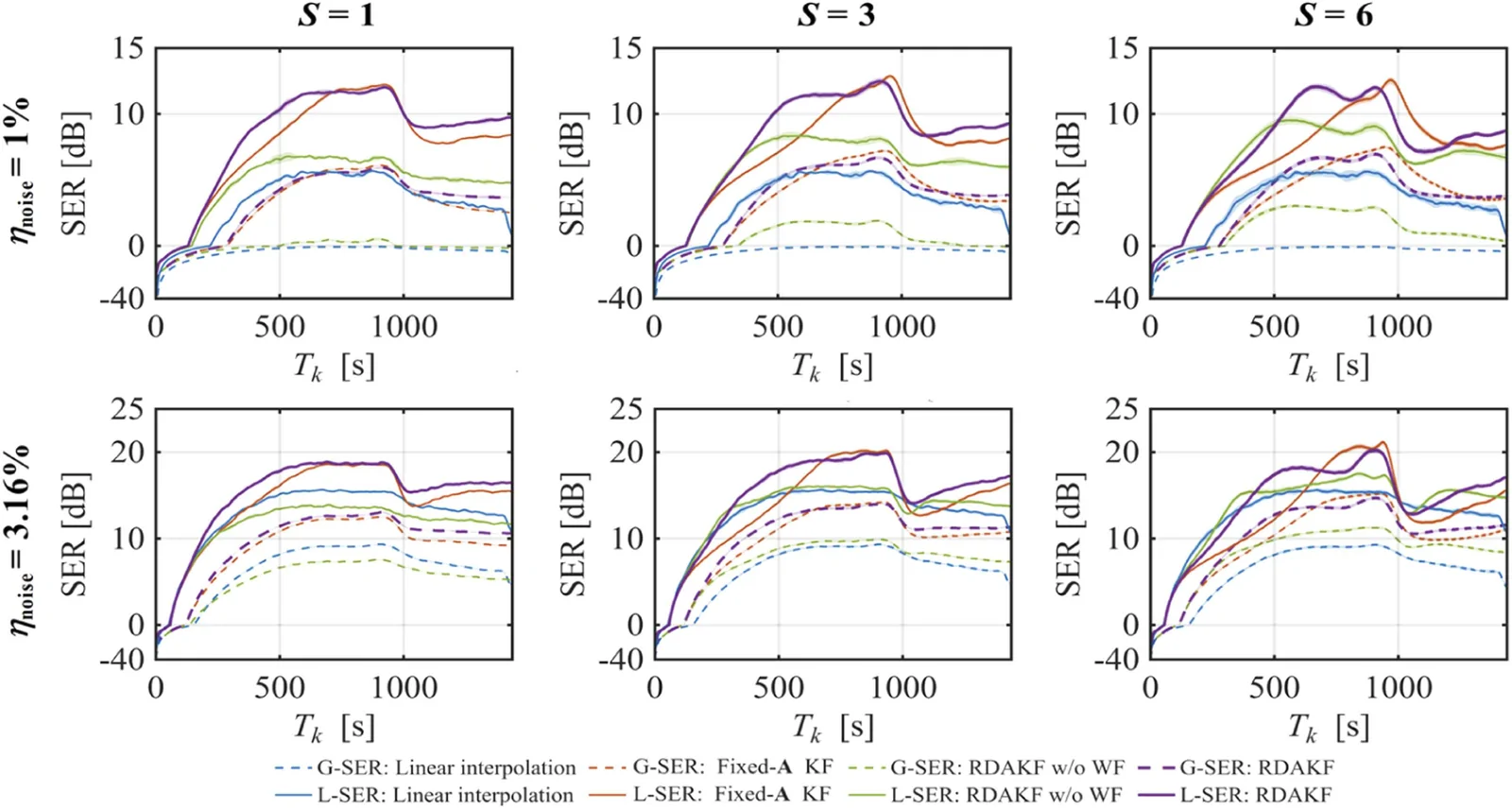
Comparison of signal recovery performance under different sparse sampling configurations and noise levels. The top row corresponds to $\eta_{\mathrm{noise}}=1\%$, and the bottom row corresponds to $\eta_{\mathrm{noise}}=3.16\%$. From left to right, the columns show results for S= 1,3 and 6, respectively. Dashed curves denote global signal-to-error ratio (G-SER), and solid curves denote local signal-to-error ratio (L-SER). Different colors represent linear interpolation, fixed-state-transition-matrix Kalman filtering (fixed-**A** KF), RDAKF without neighborhood-weighted transition fusion (RDAKF w/o WF), and the full RDAKF configuration. Each curve represents the mean over five independent noise realizations, and the shaded bands indicate ±1 standard deviation (SD) across these realizations.

Fig. 2(e) shows the temporal trajectory at detection location 28 and waveform time t = 14.2 μs, rather than an average over the full field. Linear interpolation exhibited relatively large fluctuations because its estimates were determined directly by adjacent sparse observations and were therefore sensitive to measurement noise. The identity transition matrix in fixed-**A** KF caused the state estimate to remain near its most recently updated value until a new observation became available. Although this behavior suppressed recursive overshoot, it produced a step-like trajectory, temporal delay, and loss of detail during nonstationary variations. RDAKF w/o WF adapted the local transition matrix whenever the location was sampled; during long unsampled intervals, however, prediction relied recursively on the retained historical model. If that model was affected by measurement noise or no longer representative of the current dynamics, errors accumulated and could produce overshoot, particularly at the higher noise level. Additional evaluations of abrupt, multi-peak, and sinusoidal dynamics for RDAKF without neighborhood transition fusion ($\gamma_{A}=$ 0) and with nonzero fusion strengths are provided in Supplementary Note S2.8 and Fig. S7; these results further support the stabilizing role of neighborhood transition fusion.

The evolution of G-SER and L-SER over the complete acquisition sequence (Fig. 3) confirmed the representative results in Fig. 2. Full RDAKF maintained comparatively high and stable recovery across sparse-sampling configurations and noise levels, indicating that its performance was not confined to selected time points or a single local trajectory. At $\eta_{\mathrm{noise}}=$ 3.16%, differences among methods were more pronounced. Linear interpolation yielded lower G-SER and L-SER because noise propagated across interpolated intervals. RDAKF w/o WF also performed worse overall than fixed-**A** KF, showing that recursive propagation of an imperfect historical transition model can introduce additional error without neighborhood fusion. Fixed**-A** KF achieved competitive global recovery during some intervals, but its local dynamic recovery remained constrained by the fixed transition model. Full RDAKF maintained competitive or superior G-SER and consistently high L-SER for S= 1, 3, and 6.

Around $T_{k}\;\approx$ 900 s, both G-SER and L-SER decreased for all methods as the abrupt change in Target 1 caused the differential PA waveforms to vary rapidly over a short interval, increasing the difficulty of prediction and missing-data recovery. The decline was more pronounced for Kalman-filter-based methods, consistent with recursive prediction from the preceding state estimate and the need for progressive correction after the change. Nevertheless, under the limiting condition S=1, full RDAKF maintained the highest G-SER and L-SER throughout the transition, indicating that neighborhood-weighted transition fusion reduced transient performance loss under extreme undersampling.

The effect of the number of sampled locations per pattern was evaluated using the same estimation method and noise level. Larger S resulted in longer pattern acquisition times and lower pattern-level update rates. For S>1, the sequential acquisition of locations within a pattern introduced finite temporal offsets relative to the pattern-completion time $T_{k}$. As shown by the temporal amplitude profiles in Fig. 2(e), larger S was associated with increased temporal smoothing, weaker transient responses, and temporal delay. Thus, S=1 provided the highest pattern-level temporal responsiveness, whereas S= 3 and S= 6 provided more direct observations per pattern at the cost of greater temporal aggregation and weaker transient capture.

Under the most severe undersampling condition (S=1), Fig. 4 compares five sparse-data processing and reconstruction strategies for dynamic QPAT. Except for direct sparse reconstruction with temporal total-variation regularization (Sparse-TTV), the other four methods first recovered spatially complete differential PA waveforms and then supplied them to the same PDE-constrained QPAT framework. Sparse-TTV instead used the sparse differential observations directly and promoted temporal consistency by penalizing the temporal difference $R_{t,k}$ between absorption-coefficient perturbations at the (k−1)-th and k-th consecutive acquisition steps:

(36) $$R_{t,k}={\left\|\Delta \mu_{a,k}-\Delta \mu_{a,k-1}\right\|}_{1},$$

where $\Delta \mu_{a,k}$ and $\Delta \mu_{a,k-1}$ are the absorption-coefficient perturbation vectors reconstructed at adjacent sparse-pattern completion times $T_{k}$ and $T_{k-1}$, respectively, and $∥\cdot {∥}_{1}$ denotes the $L_{1}$ norm. Temporal TV is implemented through a proximal update using relative-step thresholding. At the l-th iteration, the temporally regularized estimate is

(37) $$\Delta \mu_{a,k}^{\left(l+1\right)}=\Delta \mu_{a,k-1}+S_{\theta_{t,k}^{\left(l\right)}}\left(\Delta \mu_{a,k}^{\left(l\right)}+\Delta \mu_{a,k,\mathrm{DC}}^{\left(l\right)}-\Delta \mu_{a,k-1}\right),$$

where $\Delta \mu_{a,k,\mathrm{DC}}^{\left(l\right)}$ denotes the data-consistency update at reconstruction iteration l, and $S_{\theta_{t,k}^{\left(l\right)}}(∙)$ is the element-wise soft-thresholding operator. For an arbitrary input vector v**,** the operator is defined as $S_{\theta }(v)=\mathrm{sign}(v)⊙\max(|v|-\theta ,0)$. Consecutive-frame differences with magnitudes below the threshold are set to zero, whereas those with magnitudes exceeding the threshold retain their sign and are reduced in magnitude by the threshold. In relative-step thresholding, the proximal threshold $\theta_{t,k}^{\left(l\right)}$ is defined adaptively as

(38) $$\theta_{t,k}^{\left(l\right)}=\mathrm{clip}\left[\lambda_{t}Q_{0.95}\left(\left|\Delta \mu_{a,k,\mathrm{DC}}^{\left(l\right)}\right|\right),\theta_{t,\min},\theta_{t,\max}\right],$$

where $\lambda_{t}$ is the relative temporal-threshold factor, $Q_{0.95}(\cdot )$ denotes the 95th percentile of the element-wise update magnitudes, and clip(⋅) restricts the threshold to prescribed lower and upper bounds. The relative factor and threshold bounds were set to $\lambda_{t}=$ 0.1, $\theta_{t,\min}=$ 1 × 10^−6^ ${\mathrm{mm}}^{-1}$, and $\theta_{t,\max}=$ 2 × 10^−4^ ${\mathrm{mm}}^{-1}$, respectively.

**Fig. 4:**
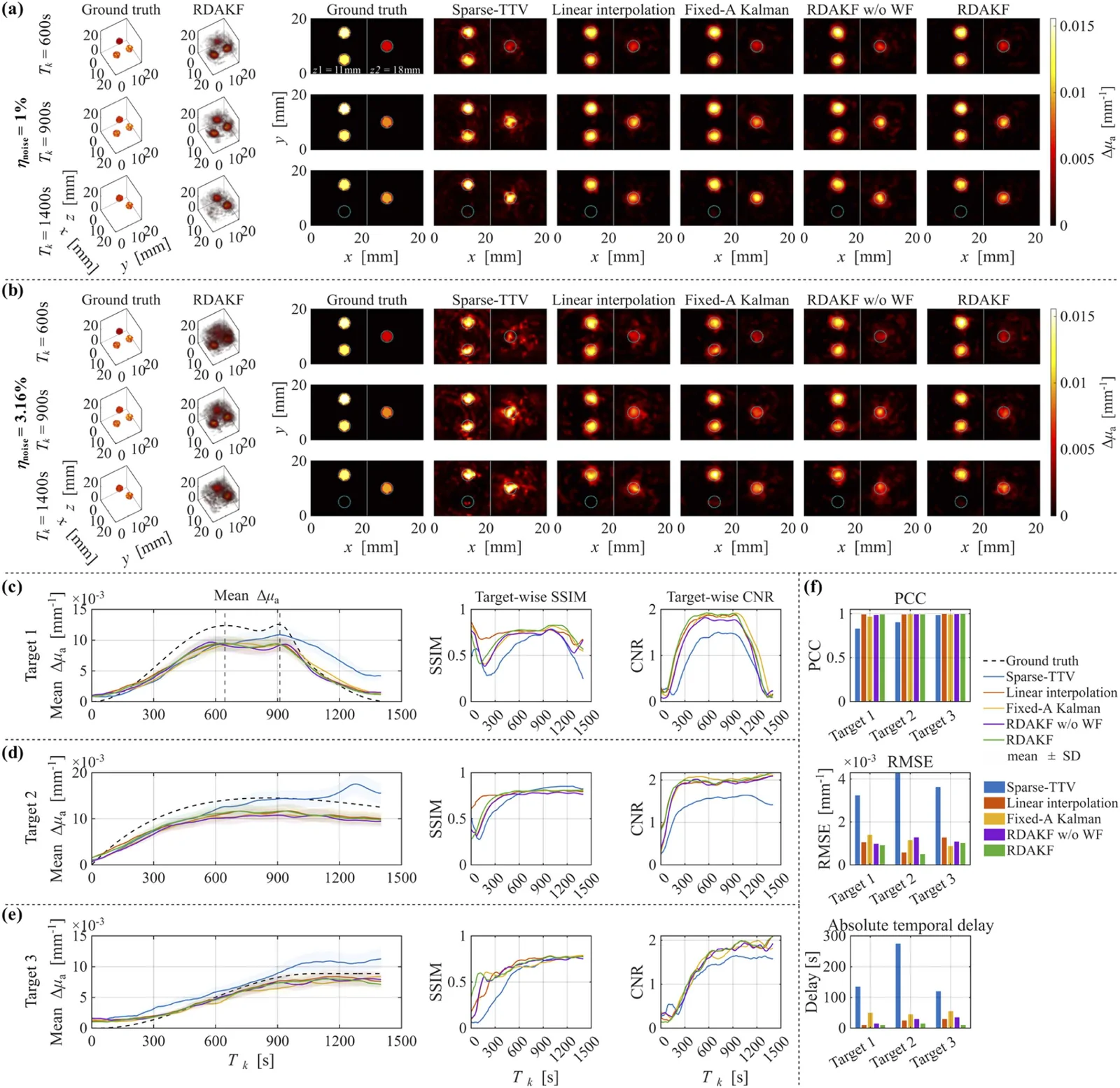
Dynamic quantitative photoacoustic tomography (QPAT) reconstruction and quantitative comparison under the limiting single-location-per-pattern configuration (S=1) at two noise levels. (a, b) Three-dimensional renderings and representative cross-sectional maps of $\Delta \mu_{a}$ at $T_{k}$ = 600, 900, and 1400 s for $\eta_{\mathrm{noise}}=$ 1% and 3.16%, respectively. Three-dimensional results compare the ground truth with full residual-driven adaptive Kalman filter (RDAKF) reconstruction; cross-sectional maps compare the ground truth, direct sparse reconstruction with temporal total-variation regularization (Sparse-TTV), linear interpolation, fixed-state-transition-matrix Kalman filtering (fixed-A KF), RDAKF without neighborhood-weighted transition fusion (RDAKF w/o WF), and full RDAKF. Circles indicate predefined target regions of interest (ROIs). All maps use the same color scale, with $\Delta \mu_{a}$ expressed in mm^−1^. (c–e) Quantitative results for Targets 1–3 at $\eta_{\mathrm{noise}}=$ 1%. From left to right, each row shows the temporal profile of mean reconstructed $\Delta \mu_{a}$, structural similarity index (SSIM), and contrast-to-noise ratio (CNR). At each frame, the mean and spatial standard deviation (SD) were calculated over voxels within the target ROI whose reconstructed values exceeded 50% of the local maximum. Dashed black curves denote ground truth, colored curves denote reconstructed results, and shaded bands indicate the mean ± SD. (f) Pearson correlation coefficient (PCC), root-mean-square error (RMSE), and absolute temporal delay for the three targets at $\eta_{\mathrm{noise}}=$ 1%. Higher PCC, lower RMSE, and smaller delay indicate better temporal agreement, quantitative accuracy, and temporal tracking, respectively.

All methods used the same forward model, reconstruction step size, maximum iteration count, warm-start strategy, and stopping criterion. Each frame was reconstructed for at most 30 iterations with step size σ= 0.02. Iterations were terminated when the mean relative decrease in the normalized data residual over the preceding three iterations was below 2 × 10^−3^ per iteration, provided that the residual did not increase. For each frame after the first, 60% of the converged reconstruction from the preceding frame was used as the initial estimate. The simulations required approximately 14 iterations per frame on average.

Fig. 4(a, b) compares ground-truth absorption-coefficient perturbations with reconstructed distributions at $T_{k}=600$, 900, and 1400 s. Sparse-TTV produced pronounced background artifacts and spurious high-intensity responses at $\eta_{\mathrm{noise}}=$ 1% because it reconstructed directly from sparse differential observations. Linear interpolation reduced these artifacts but retained diffuse background responses, target broadening, and intensity distortion, particularly at $T_{k}=$ 900 and 1400 s. Fixed**-**A KF and RDAKF w/o WF further improved target visibility, although residual non-target responses and deviations in target amplitude and spatial distribution remained. Full RDAKF most accurately recovered target locations, compact distributions, and relative intensity evolution across the representative time points. At the higher noise level $\eta_{\mathrm{noise}}=$ 3.16%, background contamination and spatial distortion increased for Sparse-TTV and linear interpolation, whereas full RDAKF retained clear target localization with only a moderate increase in background response. These findings are consistent with the waveform-recovery results: interpolation error, fixed-A model mismatch, and local overshoot propagated into the quantitative inversion, whereas neighborhood-weighted transition fusion reduced this error propagation and improved reconstruction stability under extreme undersampling.

Fig. 4(c–f) quantitatively compares the five reconstruction pipelines under S= 1 and $\eta_{\mathrm{noise}}=$ 1%. Metrics were evaluated separately for each target. For the mean $\Delta \mu_{a}$ curves in Fig. 4(c–e), voxels within each fixed ground-truth target region whose reconstructed values exceeded 50% of the local maximum were retained at each frame, and their mean and standard deviation were calculated. Target-wise structural similarity index (SSIM) was evaluated within a fixed three-dimensional region formed by isotropically expanding the corresponding ground-truth target region by 1 mm. Reconstructed (RC) and ground-truth (GT) distributions were extracted from this region, and SSIM was calculated as

(39) $${\mathrm{SSIM}}_{j,k}=\frac{\left(2\mu_{j,k}^{\mathrm{RC}}\mu_{j,k}^{\mathrm{GT}}+C_{1}\right)\left(2\sigma_{j,k}^{\mathrm{RC},\mathrm{GT}}+C_{2}\right)}{\left[{\left(\mu_{j,k}^{\mathrm{RC}}\right)}^{2}+{\left(\mu_{j,k}^{\mathrm{GT}}\right)}^{2}+C_{1}\right]\left[{\left(\sigma_{j,k}^{\mathrm{RC}}\right)}^{2}+{\left(\sigma_{j,k}^{\mathrm{GT}}\right)}^{2}+C_{2}\right]},$$

where $\mu_{j,k}^{\mathrm{RC}}$ and $\mu_{j,k}^{\mathrm{GT}}$ are the mean RC and GT values within the evaluation region; ${\left(\sigma_{j,k}^{\mathrm{RC}}\right)}^{2}$ and ${\left(\sigma_{j,k}^{\mathrm{GT}}\right)}^{2}$ are the corresponding variances; and $\sigma_{j,k}^{\mathrm{RC},\mathrm{GT}}$ is their covariance. The stabilizing constants are $C_{1}={\left(0.01\rho \right)}^{2}$ and $C_{2}={\left(0.03\rho \right)}^{2}$, where ρ is the common dynamic range used for all methods. SSIM values closer to 1 indicate greater structural agreement. Target-wise contrast-to-noise ratio (CNR) used the fixed GT target region and a background region obtained by excluding all targets from the evaluation domain. CNR was calculated as

(40) $${\mathrm{CNR}}_{j,k}=\frac{\left|\mu_{T,j,k}^{\mathrm{RC}}-\mu_{B,k}^{\mathrm{RC}}\right|}{\sqrt{{\left(\sigma_{T,j,k}^{\mathrm{RC}}\right)}^{2}+{\left(\sigma_{B,k}^{\mathrm{RC}}\right)}^{2}}},$$

where $\mu_{T,j,k}^{\mathrm{RC}}$ and $\mu_{B,k}^{\mathrm{RC}}$ are the mean reconstructed values in the target and background regions, and $\sigma_{T,j,k}^{\mathrm{RC}}$ and $\sigma_{B,k}^{\mathrm{RC}}$ are the corresponding standard deviations. Higher CNR indicates better target-to-background separability. Pearson correlation coefficient (PCC), root-mean-square error (RMSE), and absolute temporal delay in Fig. 4(f) were calculated from the target-specific mean $\Delta \mu_{a}$ temporal profiles. PCC was evaluated between the reconstructed and ground-truth mean $\Delta \mu_{a}$ profiles over the common interval; values closer to 1 indicate stronger temporal agreement. RMSE was calculated over the complete evaluation interval and measures amplitude error. Absolute delay $\tau_{j}$ was obtained by lagged-correlation analysis from the integer lag $u_{j}$ that maximized the PCC between reconstructed and ground-truth profiles as

(41) $$u_{j}=\arg\underset{u\in U}{\max}\mathrm{PCC}\left[m_{j}^{\mathrm{RC}}(k+u),m_{j}^{\mathrm{GT}}(k)\right],\;\tau_{j}=\left|u_{j}\right|\Delta T,$$

where $m_{j}^{\mathrm{RC}}$ and $m_{j}^{\mathrm{GT}}$ are the reconstructed and ground-truth mean-$\Delta \mu_{a}$ temporal profiles, respectively. U is the predefined lag set, and ΔT is the sparse-pattern acquisition interval.

The temporal profiles in the left column of Fig. 4(c–e) were smoothed with a 21-point moving-median filter for visualization; all metrics in Fig. 4(f) were calculated from unsmoothed data. Sparse-TTV directly reconstructed from sparse observations and therefore exhibited pronounced amplitude error and temporal fluctuation. Linear interpolation recovered the overall trend but estimated missing states using observations acquired both before and after the missing time point, making it noncausal. Fixed**-**A KF suppressed noise effectively, but its time-invariant transition model smoothed and delayed rapid variations, particularly the two local peaks of Target 1. RDAKF w/o WF improved tracking through residual-driven transition adaptation, but overshoot and peak shifts remained during intervals without direct observations. Full RDAKF added neighborhood-weighted transition fusion, reducing local overshoot and its propagation into quantitative reconstruction; its temporal profiles most closely followed the GT. Kalman-filter-based estimates depend only on current and past observations, making the RDAKF signal-estimation stage causal and more suitable for online implementation than the interpolation approach.

The target-wise SSIM results in Fig. 4(c–e) show that linear interpolation performed comparatively well during the initial period, before sufficient direct observations had accumulated for RDAKF to adapt the local transition dynamics. As observations accumulated, full RDAKF generally achieved higher SSIM than linear interpolation and provided more stable structural preservation. CNR showed a similar pattern: full RDAKF achieved the highest or near-highest values over most of the dynamic interval, indicating effective suppression of background fluctuations while preserving target responses. Sparse-TTV and linear interpolation were degraded by background artifacts, fixed-A KF weakened target contrast through excessive smoothing during rapid changes, and RDAKF w/o WF remained susceptible to local overshoot. These effects were more pronounced at $\eta_{\mathrm{noise}}=$ 3.16% (Supplementary Note S3.1 and Fig. S8(a–c)).

Although all methods achieved high PCC values in Fig. 4(f), RMSE and absolute delay differentiated their performance more clearly. High PCC indicates recovery of the overall temporal trend but does not ensure accurate amplitude or temporal localization. Fixed**-**A KF showed the largest delay, approaching 300 s for Target 1, consistent with the limited responsiveness of its time-invariant transition model to rapid local changes. Linear interpolation produced a small delay but a larger RMSE because amplitude and interpolation errors propagated into the inversion. RDAKF w/o WF improved tracking, although local overshoot increased quantitative error at some time points. At $\eta_{\mathrm{noise}}=$ 1%, full RDAKF achieved the lowest or near-lowest RMSE for all targets while maintaining small delay. Under the higher-noise condition $\eta_{\mathrm{noise}}=$ 3.16% (Supplementary Note S3.1 and Fig. S8), full RDAKF retained high temporal correlation and low reconstruction error, with a modest increase in delay. This behavior reflects a noise–responsiveness trade-off: stronger model-based prediction, covariance regulation, and transition fusion suppress noise-induced fluctuations but may modestly delay the response.

To further evaluate the AER framework under heterogeneous dynamics and the limiting sparse-sampling configuration S=1, Fig. 5 considers an abrupt absorption change, a periodic sinusoidal variation, and slow target displacement. Target 1 underwent a step-like change, Target 2 completed two sinusoidal cycles**,** and Target 3 translated laterally before returning to its initial position while maintaining a constant prescribed absorption coefficient (Fig. 5(a–c)).

**Fig. 5:**
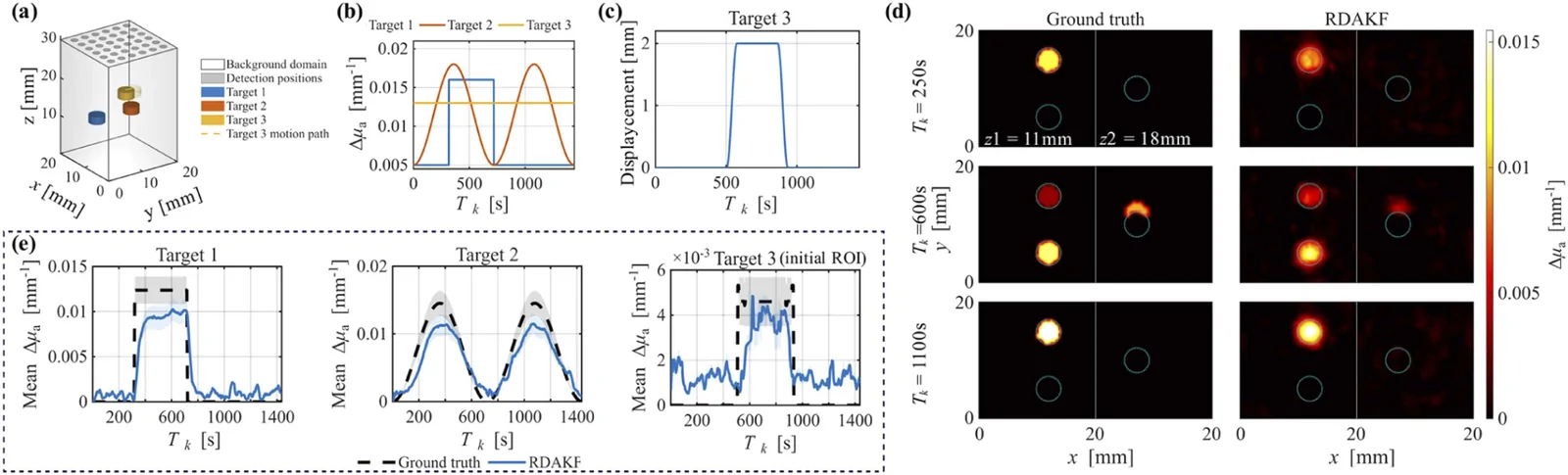
Residual-driven adaptive Kalman filter (RDAKF)-based dynamic quantitative photoacoustic tomography (QPAT) under heterogeneous temporal and motion-induced variations at $\eta_{\mathrm{noise}}=1\%$. (a) Three-dimensional phantom geometry, target locations, detection positions, and prescribed trajectory of Target 3. (b) Prescribed absorption profiles: a step-like change for Target 1, a two-cycle sinusoidal variation for Target 2, and a constant level for Target 3. (c) Prescribed displacement of Target 3, comprising a transient 2-mm translation followed by a return to its initial position. (d) Ground-truth and RDAKF-reconstructed absorption-coefficient perturbation maps at $T_{k}=$ 250, 600 and 1100 s . Cyan circles indicate initial target regions. (e) Temporal profiles of mean reconstructed absorption-coefficient perturbation. At each frame, the mean and spatial standard deviation (SD) were calculated over voxels exceeding 50% of the frame-specific maximum within each target region of interest (ROI); the initial ROI was used for Target 3. Its apparent temporal variation resulted from finite-element node assignment and mesh-to-grid interpolation during motion. Dashed black curves denote ground truth, blue curves denote reconstructed means, and shaded bands denote spatial SDs.

As shown in Fig. 5(d), the AER framework recovered the principal target locations and temporal variations and captured the displacement of Target 3. The 50%-thresholded mean absorption-coefficient perturbations tracked the abrupt change of Target 1 and the periodic response of Target 2 (Fig. 5(e)). For Target 3, apparent variation within the initial ROI arose from discrete finite-element node assignment and mesh-to-grid interpolation during motion rather than from a prescribed absorption change; nevertheless, the reconstructed trajectory remained consistent with the ground-truth trend. The three target-specific responses remained qualitatively separable without evident inter-target interference. These results support the applicability of the framework to nonstationary absorption changes and slow spatial motion.

An additional simulation evaluated heterogeneous periodic dynamics under highly sparse spatial sampling. Among 36 candidate locations, only one was sampled at each acquisition step (S= 1). The three targets completed 2, 3, and 4 sinusoidal cycles over 1440 s. RDAKF recovered their locations and periodic absorption variations. As temporal frequency increased, PCC decreased and RMSE increased, indicating a gradual loss of accuracy for faster dynamics. Absolute delays were approximately 30, 25, and 30 s and did not increase monotonically with temporal frequency. Detailed results are provided in Supplementary Note S3.2 and Figs. S9–S10.

To evaluate the computational cost of the estimation–reconstruction pipeline under the simulation conditions, Table 1 summarizes representative computation times and key temporary-memory footprints for RDAKF signal recovery and QPAT reconstruction. The benchmark used 36 detection locations, 281 data points per PA waveform, an optical finite-element mesh with 105895 nodes, and a 40 × 40 × 60 k-Wave grid.

**Table 1:** Representative computation times and key temporary-memory footprints for RDAKF signal recovery and QPAT reconstruction.

Computational module	Configuration or operation	Elapsed time	Key temporary memory
RDAKF signal recovery	*S* = 1	25.69 ms/RDAKF update	136.51 MB
	*S* = 3	30.88 ms/RDAKF update	138.92 MB
	*S* = 6	42.60 ms/RDAKF update	142.53 MB
QPAT reconstruction	Optical forward solution	1.6067 s/iteration	7150.35 MB
	Acoustic forward solution	1.8946 s/iteration	
	Adjoint solution	2.4923 s/iteration	

Benchmarks were obtained in MATLAB R2025b on Windows 11 Pro using a 13th-generation Intel Core i7–13700 CPU, 31.75 GB RAM, and an NVIDIA GeForce RTX 4060 Ti GPU with 16 GB memory.

The heterogeneous simulation illustrates the complementary roles of the three sequentially coupled AER components. SPC acquisition cycles through the predefined sparse sampling patterns in sequence to obtain direct observations at different candidate locations; RDAKF recovers spatially complete differential PA waveforms through residual-driven transition adaptation and neighborhood-weighted transition fusion; and PDE-constrained QPAT converts the recovered waveforms into spatially resolved absorption-coefficient perturbations. Under the limiting condition S= 1, the integrated framework preserved the distinct abrupt, periodic, and motion-induced target responses without evident inter-target crosstalk and recovered the trajectory of the slowly moving target. These findings support dynamic tracking and quantitative reconstruction under controlled heterogeneous dynamics with one directly observed location per sparse pattern.

### 3.2 Dynamic phantom experiments

Tissue-mimicking flow-phantom experiments were conducted to evaluate the system and AER framework under controlled dynamic conditions. The phantom measured 25 × 25 × 30 mm³ (Fig. 6(a)) and consisted of agar mixed with India ink and 20% Intralipid to provide optical absorption and scattering, respectively. At 1064 nm, the background absorption and reduced scattering coefficients were $\mu_{a,\mathrm{bg}}\;=$ 0.014 mm^−1^ and $\mu_{s,\mathrm{bg}}^{\prime }\;=$ 1 mm^−1^. Two parallel cylindrical channels (2-mm diameter, 15-mm depth, and 2-mm edge-to-edge separation) mimicked vascular structures. The scanning region was discretized into an 8 × 8 grid of 64 locations with 3-mm center-to-center spacing. SPC patterns contained S=1, 4 and 8 locations, corresponding to mean pattern acquisition times of 1.19, 4.76, and 10.00 s, respectively.

**Fig. 6:**
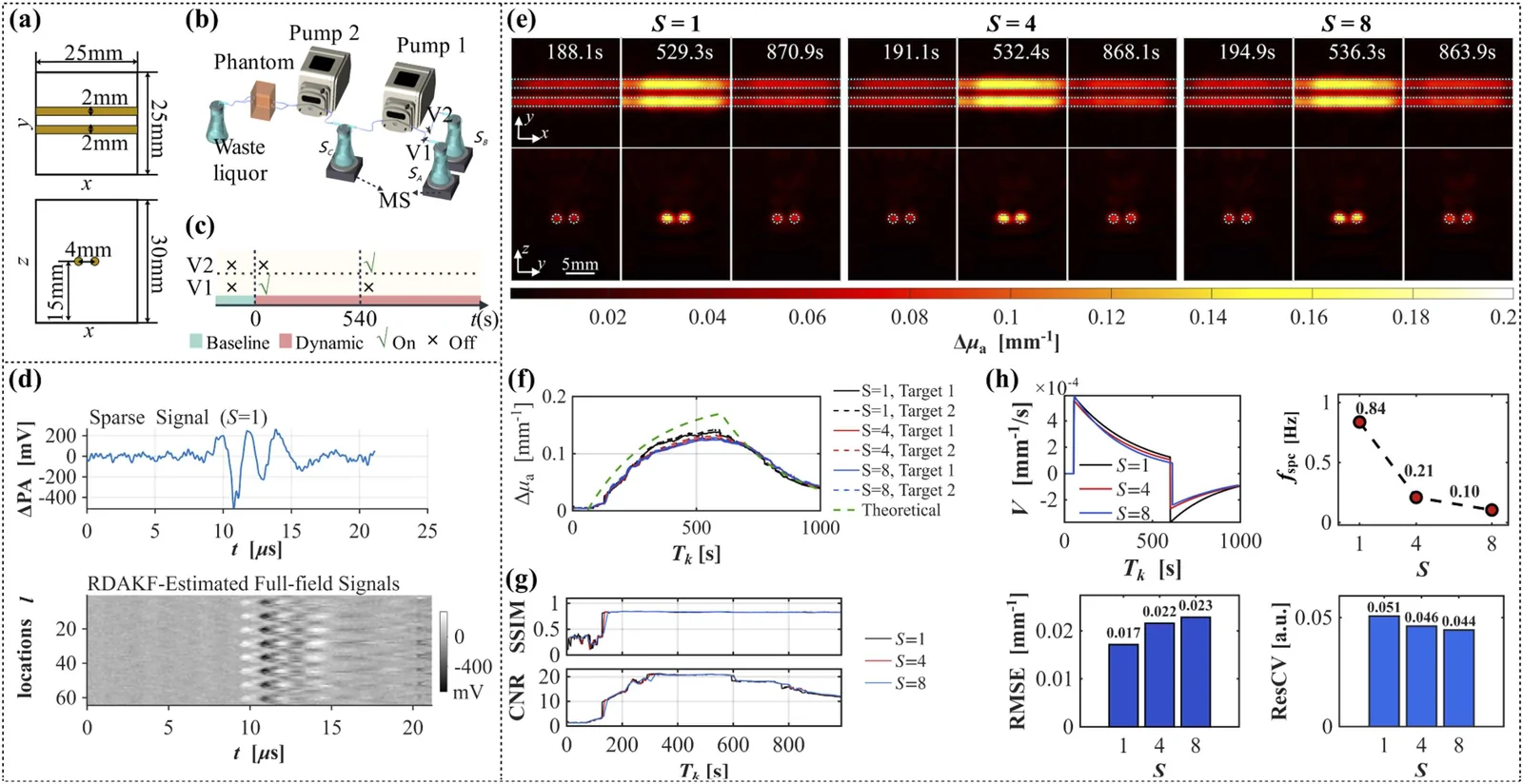
Dynamic perfusion phantom and experimental validation of the acquisition-estimation-reconstruction (AER) framework. (a) Agar-based tissue-mimicking phantom containing two parallel cylindrical channels. (b) Fluidic system for generating dynamic absorption contrast. Three solution reservoirs (*S*_*A*_, *S*_*B*_, and *S*_*C*_) are connected to two peristaltic pumps through valves V1 and V2 and placed on magnetic stirrers (MSs). (c) Valve-timing sequence; “On” and “Off” denote open and closed states for generating time-varying absorption-coefficient perturbations, $\Delta \mu_{a}$. (d) Signal recovery under sparse-pattern sampling (*S* = 1). Top: measured differential photoacoustic (PA) waveform at sampled location l = 15. Bottom: full-field waveforms estimated by the residual-driven adaptive Kalman filter (RDAKF). (e) Representative reconstructed cross-sectional maps for different S values and the corresponding sparse-pattern acquisition rates. The *x*–*y* (*z* = 15 mm) and *x*–*z* (*y* = 12.5 mm) slices are shown. (f) Temporal profiles of mean reconstructed $\Delta \mu_{a}$ and the theoretical reference, together with nonlinear fits for each *S*. At each frame, the mean was calculated over target-region voxels exceeding 50% of the local maximum. (g) Temporal evolution of structural similarity index (SSIM) within the target region and contrast-to-noise ratio (CNR) between target and background regions. (h) Quantitative comparison among sampling configurations. Top left: temporal derivatives, or evolution rates *V*, calculated from fitted $\Delta \mu_{a}$ curves; top right: mean sparse-pattern acquisition rates; bottom left: root-mean-square error (RMSE) relative to the theoretical reference; bottom right: residual coefficient of variation (ResCV) relative to the fitted temporal profiles.

Three solutions generated the dynamic absorption contrast: high-absorption solution *S*_*A*_ ($\mu_{a,A}\;=$ 0.2 mm^−1^), low-absorption solution *S*_*B*_ ($\mu_{a,B\;}=$ 0.014 mm^−1^), and a mixing reservoir *S*_*C*_ , initially containing the background solution ($\mu_{a,C}\;=$ 0.014 mm^−1^). Their reduced scattering coefficient of all solutions was fixed at $\mu_{s}^{\prime }\;=$ 1 mm^−1^, such that PA variations primarily reflected absorption changes. Before dynamic acquisition, the phantom was filled with 50 mL of *S*_*C*_ and all valves were closed to establish a stable no-flow baseline. During dynamic acquisition, two peristaltic pumps and control valves (Fig. 6(b, c)) were used to maintain balanced inflow and outflow while *S*_*A*_ and *S*_*B*_ were alternately introduced into *S*_*C*_. A flow-switching event occurred at approximately 540 s, followed by an empirically measured transport delay of approximately 60 s. The pump flow rate was 11.50 mL/min, and a magnetic stirrer operated continuously to promote homogeneous mixing and prevent sedimentation. A flow-weighted mixing model described the effective channel absorption coefficient and provided the theoretical reference trajectory. Independent photon-counting calibration assessed agreement between measured and model-predicted absorption dynamics; details are provided in Supplementary Note S4 and Figs. S11–S13.

Signal-estimation and reconstruction results for the different sparse-pattern configurations are shown in Fig. 6(d–h). RDAKF parameters were fixed at $P_{0}=$ 10, $Q_{0}=$ 0.023, $R_{0}=$ 1, $\eta_{0}=$ 0.06, and $\gamma_{A}=$ 0.6 for all phantom experiments. The stopping criterion yielded approximately 18 reconstruction iterations per frame on average. Even for *S* = 1, RDAKF recovered a spatially complete set of differential PA waveforms while preserving the dominant temporal features (Fig. 6(d)). For quantitative evaluation, the known target geometry defined target-specific ROIs. At each acquisition time, voxels within an ROI whose reconstructed $\Delta \mu_{a}$ values were at least 50% of the local peak were retained, and their mean defined the target-specific $\Delta \mu_{a}$ trajectory. Target-wise SSIM was calculated within a fixed three-dimensional evaluation region obtained by expanding the full target ROI by 1 mm in each direction; reconstructed and reference distributions were normalized independently by their positive peaks. CNR used the full target ROI and a surrounding local background, with a one-voxel dilated boundary layer excluded. SSIM and CNR in Fig. 6(g) were averaged over the two targets. Temporal evolution rate V was calculated from the first derivative of the reconstructed $\Delta \mu_{a}$ profile, and $f_{\mathrm{spc}}$ was the reciprocal of the mean acquisition time per sparse pattern. RMSE was calculated between reconstructed and reference $\Delta \mu_{a}$ trajectories over their common interval. The residual coefficient of variation (ResCV) was the standard deviation of the reconstruction residual divided by the absolute mean of the reference trajectory.

Fig. 6(e) shows that the principal spatial distribution of $\Delta \mu_{a}$ was recovered with limited geometric distortion even under severe undersampling. The corresponding SSIM and CNR trajectories are shown in Fig. 6(g). During the initial period ($T_{k}\;<$ 170 s), both metrics were low and fluctuated, particularly for S= 1, because few direct observations were available and reconstruction constraints were weak. SSIM and CNR then increased rapidly and stabilized after approximately 170 s. In the steady state, SSIM remained within 0.81–0.84, and CNR was consistently high with little variation among sampling configurations, indicating stable structural recovery and target-to-background contrast. CNR gradually decreased later in the dynamic sequence, whereas SSIM remained stable, suggesting preservation of structure as target contrast diminished.

Fig. 6(f) shows that the framework tracked mean $\Delta \mu_{a}$ during wash-in and wash-out. Compared with larger S, S=1 more closely reproduced the theoretical rising and falling slopes, indicating reduced temporal-integration effects and improved transient capture. Fig. 6(h) summarizes the associated trade-offs. Mean sparse-pattern acquisition rate, defined as the reciprocal of the mean pattern acquisition time including waveform acquisition, mechanical translation, and stage settling, decreased from 0.84 Hz at S= 1–0.10 Hz at S= 8. Accordingly, temporal evolution velocity V, which reflects responsiveness to rapid perturbations, was highest at S= 1. RMSE between reconstructed $\Delta \mu_{a}$ and the theoretical reference increased from 0.017 to 0.023 mm^−1^ with increasing S, indicating greater error from temporal integration and delay. Conversely, ResCV of the reconstructed $\Delta \mu_{a}$ trajectory decreased slightly from 0.051 to 0.044 as S increased, indicating improved numerical stability. Thus, increasing the number of locations per pattern improved stability but reduced temporal resolution and increased smoothing and delay.

An additional controlled perturbation experiment evaluated responsiveness to an abrupt change superimposed on a smoothly evolving process. Fig. 7(a) shows the phantom geometry and reconstruction plane. At approximately 300 s, a prescribed volume of the low-absorption solution $S_{B}$ was rapidly added to the continuously stirred mixture. Owing to the empirically measured reservoir-to-channel transport delay of approximately 60 s, the resulting absorption decrease reached the imaged dynamic-target channel at approximately 360 s. The original infusion condition was then maintained, allowing the absorption coefficient to recover gradually. The corresponding model-based reference trajectory was calculated using the same flow-mixing configuration and incorporated this fixed transport delay, as described in Supplementary Note S4 and shown in Fig. S13(a).

**Fig. 7:**
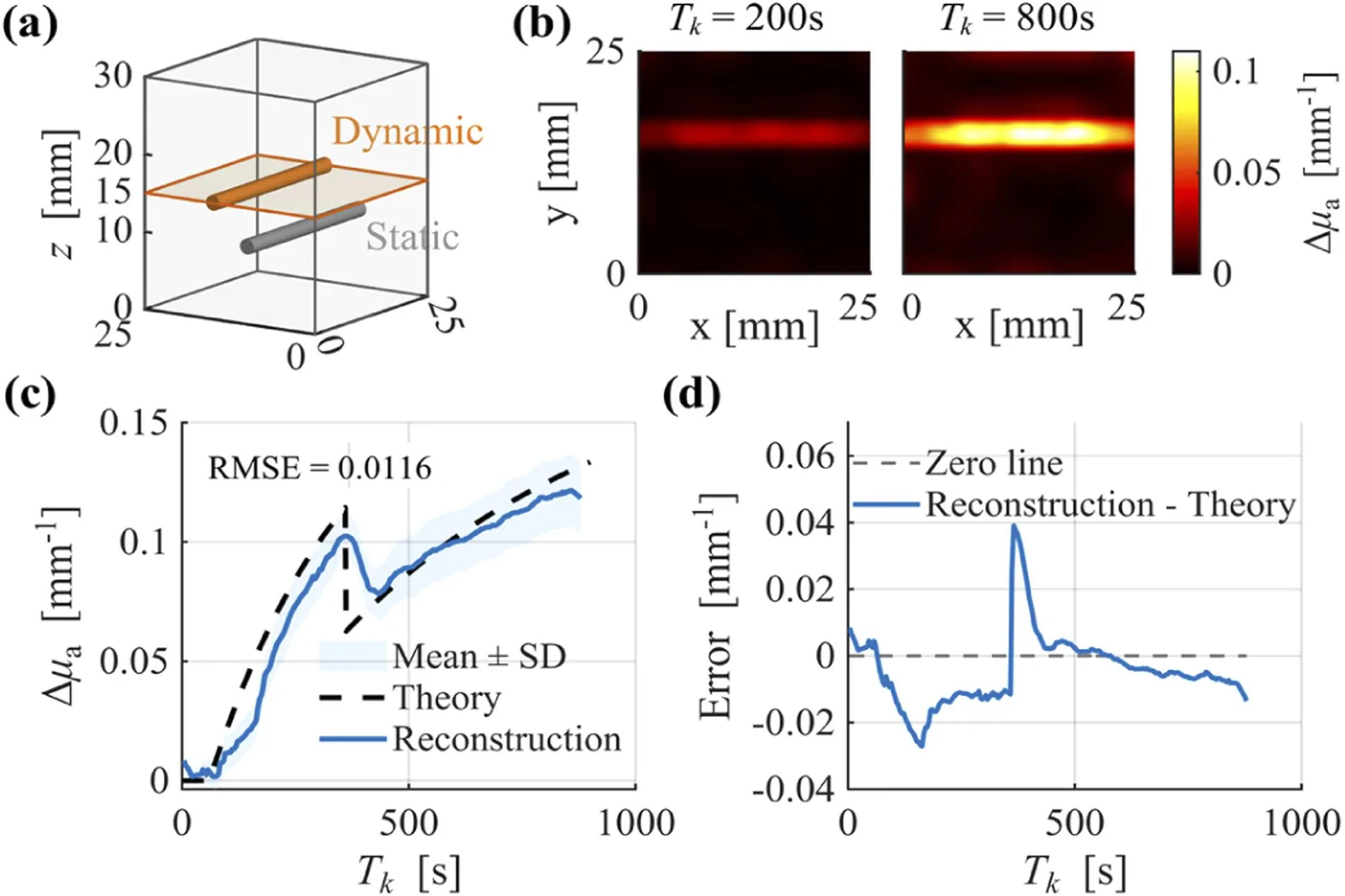
Dynamic reconstruction of the abrupt-change phantom experiment. (a) Phantom geometry with a dynamic target at y=15 mm, z= 15 mm and a static target at y=10 mm, z=10 mm. The orange plane indicates the reconstructed plane at z= 15 mm. (b) Reconstructed $\Delta \mu_{a}$ distributions at representative time points using a common color scale. (c) Mean reconstructed $\Delta \mu_{a}$ temporal trajectory within the 50%-thresholded target region compared with the theoretical reference; the shaded band indicates spatial mean ± standard deviation (SD) across the retained voxels, and the root-mean-square error (RMSE) is shown in the plot. (d) Reconstruction error, defined as the mean reconstructed $\Delta \mu_{a}$ within the same target region minus the corresponding theoretical reference at each frame.

Representative cross-sectional reconstructions are shown in Fig. 7(b). From 200–800 s, the dynamic-target response increased progressively while the surrounding background remained suppressed. The reconstructed trajectory followed the overall model-based reference and captured the abrupt absorption decrease near 360 s, with an RMSE of 0.0116 mm^−1^ relative to the theoretical curve (Fig. 7(c)). The largest discrepancy occurred during this transition, where the reconstructed response was moderately smoothed and slightly delayed, as further indicated by the reconstruction error in Fig. 7(d). The localized discrepancy remained confined to the transition interval and did not alter the recovered direction of the perturbation. Finite mixing, fluid transport, sparse-pattern acquisition, and recursive waveform estimation may all have contributed to this transient deviation. The error decreased after the transition, and the recovered trajectory returned toward the model-based reference. These results demonstrate sensitivity to a controlled abrupt perturbation following prolonged smooth evolution.

Reducing the number of locations per sparse pattern increased the pattern-level acquisition rate and reduced response delay, while spatial reconstruction quality remained comparable across the tested configurations. The framework recovered both gradual variations and the imposed abrupt perturbation, although moderate smoothing and transient error remained near rapid transitions. These results support coordinated sparse acquisition, full-field waveform estimation, and quantitative reconstruction under sparse acquisition conditions.

### 3.3 Ex vivo experiment

To evaluate quantitative monitoring in a heterogeneous deep-tissue propagation environment, an ex vivo experiment was conducted using a three-layer porcine adipose-tissue model (Fig. 8(a)). The porcine tissue was obtained commercially from a local market; no live animals were used or sacrificed specifically for this study, and institutional animal ethics approval was therefore not required. The tissue block measured approximately 50 × 50 × 28 mm³ (Fig. 8(b)). Two slightly curved silicone microtubules, representing static and dynamic inclusions, were embedded at a depth of approximately 13.5 mm; the perturbation in tube B was the quantitative target. Ultrasound coupling gel between tissue layers and on the upper surface provided acoustic transmission and detector coupling. At each sampled location, ten PA waveforms were acquired at 20 Hz and averaged, giving a nominal acquisition time of 0.5 s per location. The 40 × 40 mm² scanning region contained 100 uniformly distributed locations at 4-mm spacing. Dynamic measurements used the limiting SPC configuration (S= 1), which provided the highest tested pattern-level acquisition rate.

**Fig. 8:**
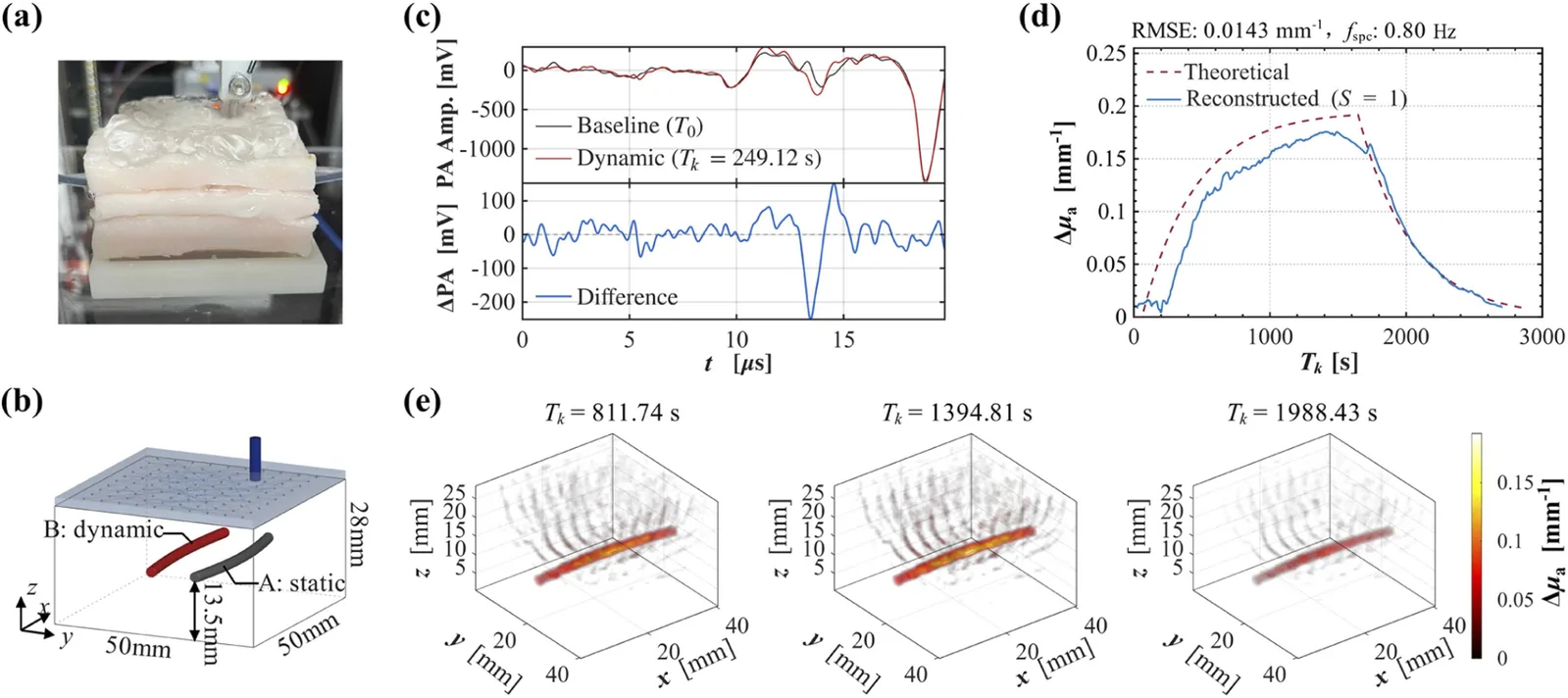
Ex vivo validation in multilayer porcine adipose tissue. (a) Experimental setup using a three-layer porcine adipose-tissue model. (b) Tissue configuration with a static high-absorption reference tube (A, 0.2 mm^-1^) and dynamically modulated tube B. (c) Representative baseline, dynamic, and differential PA waveforms at detection location 55. (d) Mean reconstructed $\Delta \mu_{a}$ temporal trajectory within the 50%-thresholded target region compared with the theoretical reference; root-mean-square error (RMSE) and mean sparse-pattern acquisition rate ($f_{\mathrm{spc}}$) are indicated. (e) Reconstructed $\Delta \mu_{a}$ distributions at representative frames.

Tube A contained India ink with a constant absorption coefficient of 0.2 mm^−1^ and served as a static high-absorption reference. Absorption in tube B was controlled dynamically by the flow system in Fig. 6(b), using the valve sequence in Fig. 6(c). At approximately 1600 s, the inflow to *S*_C_ was switched from *S*_*A*_ to *S*_B_, inducing a high-to-low absorption transition corresponding to wash-out. The peristaltic-pump flow rate was 7.67 mL/min. The effective absorption coefficient in tube B varied over the range $\mu_{a}=$0.014–0.2 mm⁻^1^, reproducing wash-in and wash-out dynamics. The multilayer tissue provided a heterogeneous ex vivo propagation environment for evaluating localization and dynamic tracking at approximately 13.5 mm depth.

Signal-estimation and reconstruction results are shown in Fig. 8(c–e). Baseline subtraction suppressed static components while preserving the waveform response to the dynamic absorption perturbation (Fig. 8(c)). At each acquisition step, the mean reconstructed $\Delta \mu_{a}$ was calculated from target-region voxels exceeding 50% of the local maximum. The resulting trajectory followed the model-based reference with an RMSE of 0.0143 mm^−1^ (Fig. 8(d)). Under *S* = 1, the mean sparse-pattern acquisition rate was 0.80 Hz. Representative reconstructions in Fig. 8(e) show that the dynamic target remained localized while its reconstructed intensity evolved with the prescribed absorption variation and the static background remained largely suppressed. The ex vivo results therefore demonstrate that the framework maintained localization and achieved model-consistent tracking through the multilayer adipose-tissue environment.

## 4 Discussion

The AER framework coordinates cyclic sparse acquisition, adaptive full-field waveform estimation, and PDE-constrained quantitative reconstruction. SPC cycles through the predefined sparse sampling patterns in sequence to provide direct observations at different candidate locations, RDAKF adapts location-specific transition matrices and propagates states at unsampled locations, and PDE-constrained inversion converts the recovered waveforms into absorption-coefficient perturbations. Baseline differencing suppresses static background contributions and focuses the inversion on dynamic absorption changes.

The method comparisons clarify the contribution of each estimation mechanism. Linear interpolation recovered overall trends but required observations acquired both before and after each missing state, precluding causal online estimation and allowing interpolation error to propagate into quantitative inversion. Fixed-**A** KF suppressed noise but introduced smoothing and delay, as its time-invariant transition model had limited responsiveness to rapid local changes. Residual-driven adaptation improved tracking in RDAKF w/o WF, but local predictions without neighborhood-weighted transition fusion remained susceptible to transient overshoot. Neighborhood-weighted transition fusion reduced these instabilities, and full RDAKF provided the most consistent balance across waveform accuracy, structural preservation, target contrast, amplitude recovery, and temporal localization. Its comparatively stable performance at the higher tested additive-noise level supports empirical robustness within the evaluated conditions.

The number of locations per pattern, S, governs a trade-off among direct spatial information, temporal fidelity, and estimation stability: larger S provides more direct observations but prolongs pattern acquisition, with the resulting within-pattern temporal offsets potentially contributing to temporal integration, smoothing, and delay, particularly when the underlying dynamics vary appreciably during one pattern acquisition, whereas smaller S increases the pattern-level acquisition rate and better preserves rapid dynamics but requires estimation at more locations. The integrated AER framework remained effective under the limiting condition S=1. In the heterogeneous simulation, abrupt, periodic, and motion-induced target responses remained qualitatively separable, and the trajectory of the slowly moving target was recovered despite its constant prescribed absorption coefficient.

Several limitations remain. First, temporal resolution is constrained by mechanical travel and positioning, as well as by the laser repetition rate. Faster scanning, reduced mechanical displacement, parallel detection, and higher-repetition-rate excitation could further increase acquisition speed.

Second, residual-driven adaptation requires sufficient observations and may show an initialization transient or temporary degradation during abrupt changes. Robust residual weighting, adaptive initialization, and change-sensitive transition updates could improve responsiveness. The present study also did not model temporally varying or spatially heterogeneous noise within a monitoring sequence. Under such nonstationary conditions, fixed process- and measurement-noise covariances may be suboptimal. Future work could combine Sage–Husa-type online covariance estimation with noise-change detection, adaptive forgetting factors, and bounded covariance constraints to update the process-noise covariance Q and measurement-noise covariance R while balancing model prediction and current observations.

Third, differential reconstruction assumes that the pre-acquired reference remains valid throughout monitoring. Reference drift or mismatch can bias both estimated differential PA waveforms and quantitative reconstruction; periodic or interleaved reference acquisition and online baseline-state estimation could mitigate this limitation. The finite-element optical model and numerical acoustic solver also impose substantial computational cost, and mismatch in sound speed, optical properties, or transducer response may reduce quantitative accuracy. Future work should investigate accelerated forward–adjoint solvers, heterogeneous acoustic and optical models, and detector-response calibration.

Overall, the AER framework mitigates the trade-off between spatial sampling density and temporal fidelity by integrating cyclic sparse acquisition, adaptive full-field waveform estimation, and physically constrained quantitative inversion. Performance under the limiting S= 1 condition supports the feasibility of dynamic tracking, preservation of heterogeneous temporal responses, recovery of slow target motion, and quantitative reconstruction with measurement-efficient mechanically scanned photoacoustic systems.

## 5 Conclusions

This study developed an AER framework for dynamic QPAT under sparse-pattern sampling. The framework integrates cyclic sparse acquisition, residual-driven full-field waveform estimation, and PDE-constrained adjoint inversion of absorption-coefficient perturbations. Numerical and experimental results showed stable dynamic tracking and quantitative reconstruction across the tested sparse-pattern configurations, including the limiting condition S=1, and comparatively stable performance at the higher tested additive-noise level. In simulation, the framework recovered abrupt, periodic, and motion-induced dynamics and tracked a slowly moving target. These findings support the feasibility of dynamic QPAT with measurement-efficient mechanically scanned systems, while highlighting the need for faster acquisition, accelerated reconstruction, improved robustness to model mismatch and experimental disturbances, and future in vivo validation.

## CRediT authorship contribution statement

**Ye Wu:** Writing – review & editing, Writing – original draft, Visualization, Software, Methodology, Investigation, Formal analysis, Data curation, Conceptualization. **Wenxing Bai:** Writing – review & editing, Visualization, Validation, Software, Methodology, Investigation, Formal analysis. **Pengfei Zhang:** Writing – review & editing, Validation, Supervision, Funding acquisition, Data curation. **Limin Zhang:** Writing – review & editing, Validation, Resources, Investigation. **Dongyuan Liu:** Writing – review & editing, Validation, Supervision, Resources, Project administration, Funding acquisition. **Feng Gao:** Writing – review & editing, Resources, Project administration, Methodology, Conceptualization, Funding acquisition.

## Ethics declaration

This study was conducted in accordance with the following guidelines for animal welfare and/or reporting: Not applicable. Only commercially obtained ex vivo porcine adipose tissue from a local market was used; no live-animal experiments were conducted. Ethics committee approval was not required. The porcine adipose tissue was commercially obtained from a local market. No live animals were used or sacrificed specifically for this study; therefore, ethics committee approval was not required. The sex of the source animal was unknown and was not relevant to the objectives of this ex vivo methodological study.

## Funding

This work was supported by the 10.13039/501100001809National Natural Science Foundation of China (Grant Nos. 62075156, 62575205, 62205239, and 12474297).

## Declaration of Generative AI and AI-assisted technologies in the writing process

During the preparation of this work, the authors used ChatGPT (OpenAI) and Gemini (Google) for language refinement and academic writing assistance. The authors reviewed and edited the content as needed and take full responsibility for the content of the published article.

## Declaration of Competing Interest

The authors declare that they have no known competing financial interests or personal relationships that could have appeared to influence the work reported in this paper.

## Data Availability

Data will be made available on request.
