# Supplementary material for: Dynamic quantitative photoacoustic tomography under sparse sampling via residual-driven Kalman filter assisted reconstruction
Source: Photoacoustics. 2026 Oct 3;52:100893. doi: 10.1016/j.pacs.2026.100893 (PMC13678021; doi:10.1016/j.pacs.2026.100893)
Supplement: Supplementary file 1 [file mmc1.docx]

**Supplementary Material**

**Dynamic Quantitative Photoacoustic Tomography under Sparse Sampling via Residual-Driven Kalman Filter Assisted Reconstruction**

# Supplementary Note S1. Timing sequence of sparse PA acquisition

As shown in Fig. S1, the translation stage first moved to the sampled location $l$, after which ten PA waveforms were acquired using ten consecutive laser pulses at 20 Hz and averaged to form one measurement. The corresponding signal-acquisition time was 0.5 s. The stage then moved to the next location $l+$1, with the translation time determined by the travel distance and prescribed stage velocity. Therefore, the actual interval between consecutive measurements depended on the sampling path.

Fig. S1. Timing sequence of PA acquisition at consecutive sampled locations. At each location, ten PA waveforms generated by ten consecutive laser pulses at 20 Hz were acquired over 0.5 s and averaged to form one measurement. The translation time between consecutive locations depended on the travel distance and the prescribed translation-stage velocity.

# Supplementary Note S2. Detailed formulation, parameter settings, validation, and computational performance of RDAKF-based signal estimation and differential QPAT reconstruction

This note complements Section 2.3 of the main text and provides an implementation-consistent description of the residual-driven adaptive Kalman filter (RDAKF). The reported implementation uses a linear full-matrix Kalman filter with a fixed identity observation matrix, location-specific prediction-residual-driven adaptation of the state-transition matrix at sampled locations, and neighborhood-weighted transition-matrix fusion at unsampled locations. The following sections define the state-space model, matrix dimensions, initialization, recursive update order, covariance scaling, parameter-selection procedure, sensitivity analyses, and computational requirements.

## S2.1. State-space representation and notation

Let $\boldsymbol{\xi}_{l}\in{\mathbb{\mathbb{R}}}^{2}$, $l=1,\ldots,L$, denote the physical coordinates of the $L$ candidate detection locations on the mechanical scanning plane. At sparse-pattern step $k$, the set of location indices in the current sparse pattern is denoted by $\Omega_{i_{k}}$. Each differential photoacoustic (PA) waveform contains $M$ data points recorded along the acoustic time-of-flight axis. Upon completion of the $k$-th sparse-pattern acquisition, RDAKF estimates the posterior differential PA waveform-state vector $\mathbf{x}_{l,k\mid k}$ at each candidate location. These location-specific posterior states are assembled into the spatially complete differential PA waveform-state matrix

|  | $\mathbf{X}_{k}\text{=}\left[ \mathbf{x}_{1,k\vert k},\mathbf{x}_{2,k\vert k},\ldots,\mathbf{x}_{L,k\vert k} \right]^{T}\text{∈}{\mathbb{\mathbb{R}}}^{M\times L}\text{.}$ | (S1) |
| --- | --- | --- |

The most recently available location-specific state-transition matrix $\mathbf{A}_{l,k-1}$ is used to propagate the posterior waveform-state estimate $\mathbf{x}_{l,k-1\mid k-1}$ from the preceding acquisition step, yielding the prior waveform-state estimate at step $k$:

|  | $\mathbf{x}_{l,k\mid k-1}=\mathbf{A}_{l,k-1}\mathbf{x}_{l,k-1\mid k-1}$. | (S2) |
| --- | --- | --- |

The associated prior state-error covariance matrix is propagated as

|  | $\mathbf{P}_{l,k\vert k-1}=\mathbf{A}_{l,k-1}\mathbf{P}_{l,k-1\vert k-1}\mathbf{A}_{l,k-1}^{T}+\mathbf{Q}$, | (S3) |
| --- | --- | --- |

where $\mathbf{P}_{l,k|k-1}\in{\mathbb{\mathbb{R}}}^{M\times M}$ denotes the prior state-error covariance at candidate location $\boldsymbol{\xi}_{l}$, $\mathbf{P}_{l,k-1|k-1}$ is the posterior state-error covariance obtained at the preceding acquisition step, and $\mathbf{Q}\in{\mathbb{\mathbb{R}}}^{M\times M}$ is the process-noise covariance accounting for model uncertainty and unmodeled waveform variations.

## S2.2. Residual-driven transition adaptation at sampled locations

For a sampled location $\boldsymbol{\xi}_{l}\in\Omega_{i_{k}}$, transition adaptation is performed before the Kalman measurement correction. The pre-adaptation model residual is defined as

|  | $\mathbf{e}_{l,k}=\mathbf{y}_{l,k}-\mathbf{H}\mathbf{x}_{l,k\vert k-1}$, | (S4) |
| --- | --- | --- |

Here, $\mathbf{y}_{l,k}$ is the current differential PA waveform observation, and $\mathbf{x}_{l,k|k-1}$ is the prior waveform estimate obtained from Eq. (S2). In the reported implementation, the observation matrix was fixed as $\mathbf{H}=\mathbf{I}_{M}$, such that adaptive modeling was concentrated on the state-transition dynamics. The pre-adaptation model residual is used only to adapt the local state-transition matrix. At the $k$-th acquisition step, the regularized prediction-error objective evaluated at the transition matrix carried forward from the preceding step is

|  | $J_{l,k}\left( \mathbf{A}_{l,k-1} \right)=\frac{1}{2}\left\Vert\mathbf{y}_{l,k}-\mathbf{H}\mathbf{A}_{l,k-1}\mathbf{x}_{l,k-1\vert k-1} \right\Vert_{2}^{2}+\frac{\lambda}{2}\left\Vert\mathbf{A}_{l,k-1} \right\Vert_{F}^{2},$ | (S5) |
| --- | --- | --- |

where $\lambda$ is the Frobenius-norm regularization coefficient. The explicit gradient evaluated at the retained transition matrix $\mathbf{A}_{l,k-1}$ is

|  | $\nabla_{\mathbf{A}_{l,k-1}}J_{l,k}=-\mathbf{H}^{T}\left( \mathbf{y}_{l,k}-\mathbf{H}\mathbf{A}_{l,k-1}\mathbf{x}_{l,k-1\vert k-1} \right)\mathbf{x}_{l,k-1\vert k-1}^{T}+\lambda\mathbf{A}_{l,k-1}.$ | (S6) |
| --- | --- | --- |

The normalized residual magnitude and the corresponding residual-dependent learning rate are defined as

|  | $r_{l,k}=\frac{\Vert\mathbf{e}_{l,k}{\Vert}_{2}}{\Vert\mathbf{y}_{l,k}{\Vert}_{2}+\epsilon}, \eta_{l,k}=\eta_{0}\frac{r_{l,k}}{c_{scale}+r_{l,k}+\epsilon},$ | (S7) |
| --- | --- | --- |

where $\eta_{0}$ is the base learning rate, $c_{\mathrm{scale}}$ is the residual-saturation constant, and $\epsilon$ is a small positive constant used to prevent division by zero. In the reported implementation, $\eta_{0}=0.06$, $c_{\mathrm{scale}}=0.8$ and the regularization coefficient was set to $\lambda={10}^{-3}$.

Following recursive prediction-error identification and normalized stochastic-gradient adaptation, the current-step transition matrix is obtained through one normalized gradient step:

|  | $\mathbf{A}_{l,k}=\mathbf{A}_{l,k-1}-\eta_{l,k}\frac{\nabla_{\mathbf{A}_{l,k-1}}J_{l,k}}{\left\Vert\nabla_{\mathbf{A}_{l,k-1}}J_{l,k} \right\Vert_{F}+\varepsilon}.$ | (S8) |
| --- | --- | --- |

The resulting $\boldsymbol{A}_{l,k}$ is directly retained as the location-specific transition matrix for the current acquisition step.

## S2.3. Full-matrix prediction and measurement correction

Using the updated current-step transition matrix $\text{A}_{\text{l}\text{,}\text{k}}$, the prior waveform state and prior state-error covariance are recomputed as

|  | ${\hat{\text{x}}}_{\text{l}\text{,}\text{k}\text{∣}\text{k}\text{−1}}\text{=}\text{A}_{\text{l}\text{,}\text{k}}{\hat{\text{x}}}_{\text{l}\text{,}\text{k}\text{−1∣}\text{k}\text{−1}},$ | (S9) |
| --- | --- | --- |
|  | $\mathbf{P}_{l,k\mid k-1}=\mathbf{A}_{l,k}\mathbf{P}_{l,k-1\mid k-1}\mathbf{A}_{l,k}^{\mathsf{T}}+\mathbf{Q}\mathbf{.}$ | (S10) |

The prior estimates initially propagated using $\text{A}_{\text{l}\text{,}\text{k}\text{-1}}$ in Eqs. (S2) and (S3) are used to evaluate the pre-adaptation model residual and determine the current-step transition matrix. The recomputed prior estimates in Eqs. (S9) and (S10), which incorporate $\text{A}_{\text{l}\text{,}\text{k}}$, are used in the subsequent Kalman measurement correction.

The Kalman innovation, innovation covariance, and Kalman gain are then given by

|  | $\boldsymbol{\nu}_{l,k}=\mathbf{y}_{l,k}-\mathbf{H}{\hat{\mathbf{x}}}_{l,k\mid k-1},$ | (S11) |
| --- | --- | --- |
|  | $\mathbf{S}_{l,k}=\mathbf{H}\mathbf{P}_{l,k\mid k-1}\mathbf{H}^{\mathsf{T}}+\mathbf{R},$ | (S12) |
|  | $\mathbf{C}_{l,k}=\mathbf{P}_{l,k\mid k-1}\mathbf{H}^{\mathsf{T}}\mathbf{S}_{l,k}^{-1}.$ | (S13) |

where $\mathbf{R}\in{\mathbb{\mathbb{R}}}^{M\times M}$ is the measurement-noise covariance matrix, $\mathbf{S}_{l,k}\in{\mathbb{\mathbb{R}}}^{M\times M}$ is the innovation covariance matrix, and $\mathbf{C}_{l,k}\in{\mathbb{\mathbb{R}}}^{M\times M}$ is the Kalman gain. The same initial measurement-noise covariance was assigned to all candidate locations as $\mathbf{R}=R_{0}I_{M}$, where $R_{0}$>0 is a scalar initialization parameter.

The posterior waveform state and covariance are updated as

|  | ${\hat{\mathbf{x}}}_{l,k\mid k}={\hat{\mathbf{x}}}_{l,k\mid k-1}+\mathbf{C}_{l,k}\boldsymbol{\nu}_{l,k},$ | (S14) |
| --- | --- | --- |
|  | $\mathbf{P}_{l,k\mid k}=\left( \mathbf{I}_{M}-\mathbf{C}_{l,k}\mathbf{H} \right)\mathbf{P}_{l,k\mid k-1}.$ | (S15) |

The prior and posterior covariance matrices are numerically symmetrized after propagation and correction to suppress finite-precision asymmetry. Thus, a sampled waveform is not directly assigned as the recovered state; instead, it corrects the prior prediction according to the relative uncertainties represented by $\mathbf{P}_{l,k\mid k-1}$ and $\mathbf{R}$.

## S2.4. Neighborhood-weighted transition fusion at unsampled locations

For an unsampled location $\boldsymbol{\xi}_{l}\notin\Omega_{i_{k}}$, no current observation is available, and therefore neither residual-driven transition adaptation nor Kalman measurement correction is performed. A fixed spatial-neighborhood graph was constructed from the physical scanning coordinates. At step $k$, only neighboring locations that had received at least one valid direct observation are retained in the active neighbor set $\mathcal{N}_{l,k}$. For $j\in\mathcal{N}_{l,k}$, the distance-dependent weight is defined as

|  | $w_{lj,k}=\frac{exp\left[ -\Vert\boldsymbol{\xi}_{l}-\boldsymbol{\xi}_{j}{\Vert}_{2}^{2}/\left( 2\sigma_{\mathrm{space}}^{2} \right) \right]}{\sum_{q\in\mathcal{N}_{l,k}} exp\left[ -\Vert\boldsymbol{\xi}_{l}-\boldsymbol{\xi}_{q}{\Vert}_{2}^{2}/\left( 2\sigma_{\mathrm{space}}^{2} \right) \right]}, j\in\mathcal{N}_{l,k}.$ | (S16) |
| --- | --- | --- |

where $\boldsymbol{\xi}_{l}$ and $\boldsymbol{\xi}_{j}$ are the physical coordinates of the unsampled location and its neighboring valid location, respectively; $\left\| \xi_{l}-\xi_{j} \right\|_{2}$ is their Euclidean distance on the mechanical scanning plane. The parameter $\sigma_{\mathrm{space}}$ controls the spatial decay range of the neighborhood weights. Here, q is a dummy summation index over all valid neighboring locations in the active neighbor set. The weights are normalized such that their sum over the active neighbor set equals one. A neighborhood-weighted transition matrix was then calculated as

|  | $A_{l,k}^{\mathrm{nbr}}=\sum_{j\in\mathcal{N}_{l,k}} w_{lj,k}\mathbf{A}_{j,k}.$ | (S17) |
| --- | --- | --- |

The transition matrix at the unsampled location is relaxed toward this neighbor-averaged transition matrix,

|  | $\mathbf{A}_{l,k}=\left( 1-\gamma_{A} \right)\mathbf{A}_{l,k-1}+\gamma_{A}\mathbf{A}_{l,k}^{\mathrm{nbr}}, {0\leq\gamma}_{A}\leq1.$ | (S18) |
| --- | --- | --- |

where the weight factor $\gamma_{A}$ controls the strength of transition sharing.

After neighborhood-weighted transition fusion, the prior waveform state and prior state-error covariance are propagated as

|  | $\mathbf{x}_{l,k\vert k-1}=\mathbf{A}_{l,k}\mathbf{x}_{l,k-1\vert k-1}$, | (S19) |
| --- | --- | --- |
|  | $\mathbf{P}_{l,k\vert k-1}=\mathbf{A}_{l,k}\mathbf{P}_{l,k-1\vert k-1}\mathbf{A}_{l,k}^{T}+\mathbf{Q}\mathbf{.}$ | (S20) |

Because no current measurement is available at the unsampled location, no Kalman correction is performed, and the prior estimates are retained as the posterior estimates:

|  | $\mathbf{x}_{l,k\mid k}=\mathbf{x}_{l,k\mid k-1}$, $\mathbf{P}_{l,k\vert k}=\mathbf{P}_{l,k\vert k-1}$. | (S21) |
| --- | --- | --- |

After each sparse-pattern acquisition, the posterior states are assembled as

|  | $\mathbf{X}_{k}=\left[ \mathbf{x}_{1,k\vert k},\mathbf{x}_{2,k\vert k},\ldots,\mathbf{x}_{L,k\vert k} \right]^{T}\in{\mathbb{\mathbb{R}}}^{M\times L}$, | (S22) |
| --- | --- | --- |

thereby forming a spatially complete differential PA waveform set for subsequent PDE-constrained dynamic QPAT reconstruction. Both the state-transition matrices and the state-error covariance matrices are retained in full $M\times M$ form, allowing coupling among acoustic time-of-flight samples to be represented during state propagation and their uncertainty correlations to be preserved during measurement correction. The initialization of $\mathbf{P}$, $\mathbf{Q}$, and $\mathbf{R}$, together with the associated parameter-selection and validation procedures, is detailed in Supplementary Note S2.7. The retained parameter settings are summarized in Table S3, and the corresponding sensitivity and validation results are presented in Figs. S2–S5.

## S2.5. Initialization

At all candidate locations, the state, transition matrix, and covariance matrices are initialized as

|  | $\mathbf{x}_{l,0\mid0}=\mathbf{0}, \mathbf{A}_{l,0}=a_{0}\mathbf{I}_{M},$ | (S23) |
| --- | --- | --- |
|  | $\mathbf{P}_{l,0\mid0}=P_{0}\mathbf{I}_{M}, \mathbf{Q}=Q_{0}\mathbf{I}_{M}, \mathbf{R}=R_{0}\mathbf{I}_{M}.$ | (S24) |

Here, $a_{0}$ is the transition initialization coefficient and $P_{0}$, $Q_{0}$, and $R_{0}$ are covariance-scale parameters. Because the initial waveform dynamics are unknown, no location-specific transition prior is imposed. In this study, $a_{0}$ was set to 1, giving $\mathbf{A}_{l,0}=\mathbf{I}_{M}$. This identity initialization corresponds to a temporal-persistence assumption before sufficient direct observations become available. Although the transition matrices are initialized identically, they subsequently evolve independently through local residual-driven adaptation at sampled locations and neighborhood-weighted transition fusion at unsampled locations.

The covariance matrices are initialized as scaled identity matrices but are retained and propagated as full matrices during recursive estimation. The parameter $P_{0}$ controls the initial state-estimation uncertainty, $Q_{0}$ controls the allowable state-model variation, and $R_{0}$ represents the initial measurement uncertainty. Their condition-specific values and validation are described in Section S2.7.

## S2.6. Recursive update order and full-field recovery

The complete update at sparse acquisition step $k$ follows the sequence below:

(1) Read the posterior states, transition matrices, and covariance matrices retained from step $k-1$.
(2) At each sampled location, evaluate the pre-adaptation model residual and the explicit transition gradient.
(3) Update the current-step transition matrix through the normalized gradient step.
(4) Recompute the prior state and prior state-error covariance using the updated transition matrix, and then perform the full-matrix Kalman measurement correction and posterior covariance update.
(5) Store a common snapshot after all sampled-location updates.
(6) At each unsampled location, form the active neighbor set and calculate the neighborhood-weighted transition matrix.
(7) Fuse the neighborhood estimate with the retained local transition matrix and propagate the state and covariance without measurement correction.
(8) Assemble the posterior states from all locations into the spatially complete waveform matrix

|  | $\mathbf{X}_{k}=\left[ \mathbf{x}_{1,k\vert k},\mathbf{x}_{2,k\vert k},\ldots,\mathbf{x}_{L,k\vert k} \right]\in{\mathbb{\mathbb{R}}}^{M\times L}.$ | (S25) |
| --- | --- | --- |

Table S1. Dimensions and roles of the principal RDAKF quantities.

| **Quantity** | **Dimension** | **Role and implementation** |
| --- | --- | --- |
| $\mathbf{x}_{l,k\vert k}$ | $M\times1$ | Posterior differential PA waveform state at a candidate location |
| $\mathbf{y}_{l,k}$ | $M\times1$ | Observed differential waveform at a sampled location |
| $\mathbf{A}_{l,k}$ | $M\times M$ | Full, location-specific, adaptive transition matrix |
| $\mathbf{H}$ | $M\times M$ | Fixed identity observation matrix |
| $\mathbf{P}_{l,k\mid k}$ | $M\times M$ | Full posterior state covariance |
| $\mathbf{Q}$ | $M\times M$ | Full process-noise covariance |
| $\mathbf{R}$ | $M\times M$ | Full measurement-noise covariance |
| $\mathbf{S}_{l,k}$ | $M\times M$ | Innovation covariance |
| $\mathbf{C}_{l,k}$ | $M\times M$ | Full Kalman gain |

As SPC cyclically changes the sampled locations, each candidate position periodically receives a direct observation, a residual-driven transition update, and a Kalman correction. Between direct observations, its waveform and covariance are recursively propagated using the retained local transition dynamics and neighborhood-weighted transition fusion. Thus, the adaptive model information progressively extends throughout the scanning region, and a complete differential PA waveform set is produced after every sparse-pattern acquisition even though only $S$ locations are directly observed at that step. The recovered full-field waveforms are then used as the input to the subsequent PDE-constrained dynamic QPAT reconstruction without additional spatial averaging of waveform amplitudes.

## S2.7. Covariance scaling and parameter validation

## *S2.7.1. Roles of* $P_{0}$*,* $Q_{0}$*, and* $R_{0}$

$P_{0}$, $Q_{0}$, and $R_{0}$ are scalar covariance-scale parameters used to initialize the posterior state-error covariance, process-noise covariance, and measurement-noise covariance, respectively; they do not represent waveform amplitudes. $P_{0}$ determines the initial state-estimation uncertainty and primarily affects the transient convergence behavior. A larger $Q_{0}$ permits greater variation of the waveform state between successive sparse-acquisition steps, thereby improving responsiveness to temporal changes but potentially increasing prediction variability. In contrast, a larger $R_{0}$ assigns lower confidence to the current waveform observation, providing stronger measurement-noise suppression at the possible cost of increased temporal smoothing and delay. Because the influence of $P_{0}$ is concentrated mainly in the initial convergence stage, it was held fixed during the $Q_{0}/R_{0}$ sensitivity analyses. The parameters $Q_{0}$ and $R_{0}$ therefore jointly determine the balance between dynamic tracking responsiveness and measurement-noise suppression throughout the recursive estimation process.

## *S2.7.2. Noise-dependent adjustment of* $R_{0}$

The additive-noise level was specified by the relative RMS standard deviation $\eta_{\mathrm{noise}}$. Increasing $\eta_{\mathrm{noise}}$ from 1% to 3.16% increases the observation-noise variance by approximately one order of magnitude. Accordingly, the nominal measurement-noise covariance scale was shifted toward larger values, while the process-noise covariance remained near the stable range identified at 1% noise:

|  | $R_{0}\left( \eta_{\mathrm{noise}}=3.16\% \right)\approx10R_{0}\left( \eta_{\mathrm{noise}}=1\% \right).$ | (S26) |
| --- | --- | --- |

## *S2.7.3. Acoustic-response amplitude scaling*

To account for detector bandwidth and frequency-dependent acoustic attenuation, two clean simulated PA datasets were generated under matched phantom, optical, acoustic, scanning, and acquisition-time conditions. One dataset represented the ideal acoustic response, whereas the other incorporated a detector center frequency of 2 MHz, an 80% fractional bandwidth, and power-law acoustic attenuation

|  | $\alpha\left( f \right)=\alpha_{0}f^{\beta},$ | (S27) |
| --- | --- | --- |

where $f$ is expressed in megahertz, $\alpha_{0}=0.75 dB/\left( \mathrm{MHz}^{1.5} \mathrm{cm} \right)$, and $\beta=1.5$. Differential waveforms were constructed relative to the reference acquisition frame, and the effective acoustic-response amplitude factor was calculated as

|  | $c_{\mathrm{ac}}=\frac{\mathrm{RMS}\left( \Delta\mathbf{P}_{\mathrm{ac}} \right)}{\mathrm{RMS}\left( \Delta\mathbf{P}_{\mathrm{ideal}} \right)}.$ | (S28) |
| --- | --- | --- |

The calculation was repeated over the complete waveform map and an effective signal region defined by samples whose ideal differential amplitude exceeded 5% of the global ideal peak. The two estimates were nearly identical. The retained factor was $c_{\mathrm{ac}}=0.0421$, corresponding to an amplitude reduction of approximately 23.7-fold and an amplitude loss of approximately 27.5 dB.

Because covariance is a second-order quantity, the effective covariance scales vary with the square of the waveform-amplitude factor:

|  | $P_{0}^{'}=c_{\mathrm{ac}}^{2}P_{0}, Q_{0}^{'}=c_{\mathrm{ac}}^{2}Q_{0}, R_{0}^{'}=c_{\mathrm{ac}}^{2}R_{0}.$ | (S29) |
| --- | --- | --- |

The unprimed parameters are the raw values supplied to the implementation when automatic amplitude scaling is enabled, whereas the primed parameters are the effective covariance scales used internally. This operation scales the covariance magnitudes only; the band-limited and attenuated waveforms remain the signals processed by RDAKF.

Table S2. RMS-based acoustic-response amplitude factor.

| **Evaluation region** | $\mathbf{c}_{\mathbf{ac}}$ | **Amplitude reduction** | **Amplitude loss** | $\mathbf{c}_{\mathbf{ac}}^{\mathbf{2}}$ |
| --- | --- | --- | --- | --- |
| Global waveform map | 0.0423263 | 23.626-fold | 27.468 dB | $1.7915\times{10}^{-3}$ |
| Effective signal region | 0.0420776 | 23.766-fold | 27.519 dB | $1.7705\times{10}^{-3}$ |

## *S2.7.4. Sensitivity-analysis design and retained covariance settings*

Four validation groups were considered: baseline observation noise at $\eta_{\mathrm{noise}}=1\%$; increased observation noise at $\eta_{\mathrm{noise}}=3.16\%$; the acoustic-response condition at 1% noise; and the acoustic-response condition at 3.16% noise. RDAKF recovery was performed over the complete acquisition sequence, while parameter sensitivity was evaluated at representative acquisition steps of 300, 800, and 1400 s. Parameters were retained from the overlapping high-performance region across representative times and both global and local metrics rather than from the maximum of a single sensitivity map.

The global and local signal-to-error ratios were defined as

|  | ${G-SER}_{k}=10\log_{10}\frac{\parallel\Delta\mathbf{P}_{k}^{\mathrm{GT}}\parallel_{F}^{2}}{\parallel\Delta\mathbf{P}_{k}^{\mathrm{est}}-\Delta\mathbf{P}_{k}^{\mathrm{GT}}\parallel_{F}^{2}+\epsilon},$ | (S30) |
| --- | --- | --- |
|  | ${L-SER}_{k}=10\log_{10}\frac{\parallel\mathbf{M}_{k}\odot\Delta\mathbf{P}_{k}^{\mathrm{GT}}\parallel_{F}^{2}}{\parallel\mathbf{M}_{k}\odot\left( \Delta\mathbf{P}_{k}^{\mathrm{est}}-\Delta\mathbf{P}_{k}^{\mathrm{GT}} \right)\parallel_{F}^{2}+\epsilon},$ | (S31) |

where $\mathbf{M}_{k}$ is a ground-truth-derived mask of the dynamically responsive target region; the superscripts GT and est denote the ground-truth and estimated full-field differential PA waveform matrices, respectively; ε is a small positive constant; and the subscript F denotes the Frobenius norm. G-SER evaluates the complete detection-location–fast-time waveform map, whereas L-SER emphasizes the dynamically responsive local region.

The sensitivity maps in Figs. S2–S5 show that the recovery performance depends mainly on the relative balance between the process-noise and measurement-noise covariance scales rather than on a single isolated parameter combination. Under the baseline-noise condition ($\eta_{\mathrm{noise}}=1\%$; Fig. S2), very small $R_{0}$ values generally produced lower G-SER because excessive confidence was assigned to the noisy observations. A broad high-performance region was observed at moderate $Q_{0}$ and $R_{0}$, although the L-SER maps were more sensitive than the G-SER maps to excessively small $Q_{0}$ or large $R_{0}$. The retained values, $Q_{0}=0.011$ and $R_{0}=1$, were selected from the region that maintained consistently high global and local recovery accuracy at *k* = 300, 800, and 1400.

When the noise level increased to $\eta_{\mathrm{noise}}=3.16\%$, the high-SER region shifted toward larger $R_{0}$, whereas the favorable range of $Q_{0}$ remained comparatively similar. This trend is consistent with the approximately tenfold increase in observation-noise variance and supports the retained setting of $Q_{0}=0.011$ and $R_{0}=10$. Thus, increasing $R_{0}$ reduced the influence of the noisier measurements without requiring a comparable increase in the process-noise covariance. Under the acoustic-response conditions (Figs. S4 and S5), the effective covariance scales were reduced by $c_{\mathrm{ac}}^{2}$, but the overall topology of the sensitivity maps remained similar to that obtained without acoustic-response scaling. The retained effective $Q_{0}'$ and $R_{0}'$ values were located within the overlapping high-SER regions across representative acquisition steps and both evaluation metrics. Overall, the broad plateaus observed in Figs. S2–S5 indicate that RDAKF does not rely on a narrowly tuned covariance pair, while the noise-dependent displacement of the favorable $R_{0}$ range confirms the expected role of the measurement-noise covariance in controlling confidence in the observations.

Fig. S2. Sensitivity of RDAKF waveform recovery to the process-noise and measurement-noise covariance scales $Q_{0}$ and $R_{0}$ at $\eta_{\mathrm{noise}}=1\%$. The top and bottom rows show the global signal-to-error ratio (G-SER) and local signal-to-error ratio (L-SER), respectively, at representative acquisition-step indices $k = 300$, 800, and 1400. Higher SER values indicate better agreement between the recovered and ground-truth differential PA waveforms. The broad high-SER regions demonstrate that the recovery performance remains stable over a range of $Q_{0}/R_{0}$ combinations rather than depending on a single isolated optimum.

Fig. S3. Sensitivity of RDAKF waveform recovery to the process-noise and measurement-noise covariance scales $Q_{0}$ and $R_{0}$ at $\eta_{\mathrm{noise}}=3.16\%$. The top and bottom rows show the global signal-to-error ratio (G-SER) and local signal-to-error ratio (L-SER), respectively, at representative acquisition-step indices $k = 300$, 800, and 1400. Higher SER values indicate better agreement between the recovered and ground-truth differential PA waveforms. The broad high-SER regions demonstrate that the recovery performance remains stable over a range of $Q_{0}/R_{0}$ combinations rather than depending on a single isolated optimum.

Fig. S4. Sensitivity of RDAKF waveform recovery to the process-noise and measurement-noise covariance scales $Q_{0}$ and $R_{0}$under the acoustic-response condition at $\eta_{\mathrm{noise}}=1\%$. The top and bottom rows show the global signal-to-error ratio (G-SER) and local signal-to-error ratio (L-SER), respectively, at representative acquisition-step indices $k = 300$, 800, and 1400. Higher SER values indicate better agreement between the recovered and ground-truth differential PA waveforms. The broad high-SER regions demonstrate that the recovery performance remains stable over a range of $Q_{0}/R_{0}$ combinations rather than depending on a single isolated optimum.

Fig. S5. Sensitivity of RDAKF waveform recovery to the process-noise and measurement-noise covariance scales $Q_{0}$ and $R_{0}$under the acoustic-response condition at $\eta_{\mathrm{noise}}=3.16\%$. The top and bottom rows show the global signal-to-error ratio (G-SER) and local signal-to-error ratio (L-SER), respectively, at representative acquisition-step indices $k = 300$, 800, and 1400. Higher SER values indicate better agreement between the recovered and ground-truth differential PA waveforms. The broad high-SER regions demonstrate that the recovery performance remains stable over a range of $Q_{0}/R_{0}$ combinations rather than depending on a single isolated optimum.

As summarized in Table S3, broad high-SER regions were observed under all four validation conditions, indicating that RDAKF did not depend on a narrowly tuned covariance pair. Increasing the observation-noise level primarily shifted the favorable measurement-noise covariance scale toward larger values, whereas the stable process-noise covariance range remained comparatively similar. The acoustic-response scaling changed the covariance magnitudes by $c_{\mathrm{ac}}^{2}$but preserved the overall sensitivity-map topology. The retained settings were selected from the overlapping high-performance regions across representative acquisition steps and both global and local metrics.

Table S3. Sensitivity-derived stable ranges and retained covariance settings.

| **Condition** | $\boldsymbol{\eta}_{\mathbf{noise}}$ | **Stable region** | **Retained setting** |
| --- | --- | --- | --- |
| Baseline noise | 1% | Raw: $Q_{0}=0.007\text{−}$0.011; $R_{0}=0.5\text{−}$2.0 | Nominal: $P_{0}=10$; $Q_{0}=0.011$; $R_{0}=1$ |
| Increased noise | 3.16% | Raw: $Q_{0}=0.009\text{−}$0.013; $R_{0}=8\text{−}$12 | Nominal: $P_{0}=10$; $Q_{0}=0.011$; $R_{0}=10$ |
| Acoustic response | 1% | Effective:$Q_{0}^{'}=(1.2\text{−}2.3)\times{10}^{-5}$;  $R_{0}^{'}=(1.8\text{−}5.3)\times{10}^{-3}$ | Effective: $P_{0}^{'}=0.0177$;  $Q_{0}^{'}=1.95\times{10}^{-5}$; $R_{0}^{'}=3.54\times{10}^{-3}$ |
| Acoustic response | 3.16% | Effective: $Q_{0}^{'}=(1.77\text{−}2.66)\times{10}^{-5}$;  $R_{0}^{'}=(1.24\text{−}2.84)\times{10}^{-2}$ | Effective: $P_{0}^{'}=0.0177$;  $Q_{0}^{'}=2.30\times{10}^{-5}$; $R_{0}^{'}=1.77\times{10}^{-2}$ |

## S2.8. Sensitivity to the neighborhood transition-fusion strength

To evaluate the joint effects of the transition-adaptation base learning rate $\eta_{0}$and neighborhood transition-fusion weight $\gamma_{A}$, five independent runs were performed for each parameter combination, and the G-SER and L-SER values were averaged. As shown in Fig. S6, when $\eta_{0}=0$, varying $\gamma_{A}$ has no effect because transition adaptation is disabled. When $\eta_{0}$>0 but $\gamma_{A}=0$, both metrics decrease as $\eta_{0}$ increases, indicating that unconstrained local transition adaptation can amplify errors under sparse observations. Introducing neighborhood fusion substantially improves recovery, with the largest gain occurring as $\gamma_{A}$ increases from 0 to 0.2. For $\gamma_{A}\geq0.4$, both metrics remain high over a broad range of $\eta_{0}$, demonstrating reduced parameter sensitivity. Overall, moderate-to-strong neighborhood fusion stabilizes local transition adaptation and enables robust waveform recovery without requiring narrowly tuned parameters.

Fig. S6. Joint sensitivity of RDAKF waveform recovery to the transition-adaptation base learning rate $\eta_{0}$ and neighborhood transition-fusion weight $\gamma_{A}$. The left and right panels show the mean global signal-to-error ratio (G-SER) and mean local signal-to-error ratio (L-SER), respectively. Each value represents the mean over five independent noise realizations. Higher SER values indicate better agreement between the recovered and ground-truth differential PA waveforms. The broad high-SER regions demonstrate that RDAKF maintains stable recovery performance over a wide range of nonzero $\eta_{0}$ and $\gamma_{A}$ combinations.

The transition-fusion strength $\gamma_{A}$ controls the balance between the retained local transition dynamics and the neighborhood-weighted transition estimate. Its sensitivity was evaluated under representative dynamic scenarios involving smooth absorption changes, abrupt multistage transitions, and target motion. When neighborhood transition fusion was disabled ($\gamma_{A}=0$), the recovered signals exhibited stronger local fluctuations and transient overshoots, particularly near abrupt temporal changes and during intervals in which target motion induced rapid waveform variation. This behavior indicates that local residual-driven transition learning alone can transfer observation noise and local model-estimation errors into unsampled predictions.

To evaluate the sensitivity of RDAKF signal recovery to the neighborhood transition-fusion strength $\gamma_{A}$, the parameter was set to 0, 0.2, 0.4, 0.6, 0.8, and 1.0 while all other parameters were held fixed. The tests were conducted in three representative dynamic scenarios encompassing smooth variations, abrupt transitions, and target motion. As shown in Fig. S7, the left column presents the prescribed absorption-coefficient profiles of the three targets, whereas the middle and right columns show the recovered differential photoacoustic signals at representative spatial locations and fast-time samples.

In Fig. S7(c), Target 3 maintained a constant prescribed absorption coefficient throughout the dynamic process and therefore appears as a horizontal line in the left panel. However, the target underwent slow spatial displacement and subsequently returned to its initial position. Consequently, although its intrinsic absorption coefficient remained unchanged, its varying spatial position produced acquisition-time-dependent changes in the differential photoacoustic signals recorded at the selected detection locations. The prescribed displacement and reconstructed motion trajectory of Target 3 are illustrated in Fig. 5(a) and (c) of the main text.

When neighborhood transition fusion was disabled ($\gamma_{A}$ = 0), the recovered signals exhibited more pronounced local fluctuations and transient overshoots, particularly during intervals involving abrupt absorption changes or rapid waveform variations induced by target motion. This result indicates that, when the state-transition dynamics are updated solely from pre-adaptation prediction residuals, observation noise and local model-estimation errors can lead to excessive transition updates and propagate into signal estimates at unsampled locations.

Introducing a nonzero $\gamma_{A}$ enabled RDAKF to combine locally adapted state-transition information with neighboring estimates, thereby reducing the influence of unstable local transition updates. Compared with the unfused case, the recovered signals obtained with nonzero fusion strengths showed improved agreement with the ground truth, together with reduced local fluctuations and transient overshoots. These improvements were particularly evident near abrupt absorption transitions and during intervals in which target motion caused relatively rapid waveform changes.

Within the range $\gamma_{A}$ = 0.2-1.0, increasing the fusion strength enhanced the contribution of neighboring transition information and produced progressively smoother recovered waveforms, with further suppression of local fluctuations and transient overshoots. Nevertheless, the differences among the nonzero settings were small, indicating that once an appropriate level of neighborhood fusion was introduced, the signal-recovery performance remained stable over a broad parameter range. Further increases in the fusion strength primarily resulted in a modest improvement in smoothness rather than a substantial change in recovery accuracy.

Overall, neighborhood transition fusion improved the stability of RDAKF signal recovery in heterogeneous dynamic scenarios, while the method exhibited limited sensitivity to the exact value of $\gamma_{A}$ over a broad nonzero range. A smaller fusion strength better preserves local dynamic responsiveness, whereas a larger value provides stronger neighborhood regularization and smoother recovery. The selected value therefore offers a stable balance between local adaptive tracking and neighborhood-based smoothing.

Fig. S7. Sensitivity of RDAKF signal recovery to the neighborhood transition-fusion strength $\gamma_{A}$ under heterogeneous absorption changes and target motion. (a–c) Three representative dynamic scenarios. The left column shows the prescribed absorption-coefficient profiles of the three targets. In (c), Target 3 maintains a constant absorption coefficient throughout the dynamic process, while its spatial position varies with the sparse-pattern acquisition completion time; the corresponding motion trajectory is illustrated in Fig. 5(a) and (c) of the main text. The middle and right columns show the recovered differential photoacoustic waveforms at selected spatial locations and fast-time samples. Black dashed curves denote the ground truth, open circles denote the sparse observations, and colored curves represent the RDAKF estimates obtained with $\gamma_{A}=0,0.2,0.4,0.6,0.8$, and $1.0$. Neighborhood transition fusion suppresses local prediction fluctuations and transient overshoots, particularly near rapid temporal transitions. The relatively small differences among the nonzero fusion strengths further indicate that the method remains stable over a broad range of $\gamma_{A}$.

## S2.9. Algorithmic parameters used in the reported implementation

Table S4 summarizes the principal algorithmic parameters used in the reported RDAKF implementation. These parameters can be divided into three groups: state and covariance initialization, residual-driven transition adaptation, and neighborhood-weighted transition fusion. Except for the condition-dependent covariance scales $Q_{0}$ and $R_{0}$, the listed settings were held fixed throughout the reported analyses. The covariance parameters were selected from the overlapping high-performance regions summarized in Table S3, whereas the transition-adaptation and neighborhood-fusion parameters were determined from the sensitivity analyses described in Supplementary Note S2.8.

Table S4. RDAKF implementation settings.

| **Parameter or option** | **Value** | **Role** |
| --- | --- | --- |
| Filter model | Linear full-matrix KF | Recursive waveform estimation |
| Observation matrix | $\mathbf{H}=\mathbf{I}_{M}$ | Direct waveform observation at sampled locations |
| $a_{0}$ | 1 | Initial $\mathbf{A}_{l,0}=\mathbf{I}_{M}$ |
| $P_{0}$ | 10 | Initial posterior covariance scale |
| $Q_{0}$, $R_{0}$ | Condition-dependent | See Table S3 |
| Base learning rate $\eta_{0}$ | 0.06 | Transition adaptation rate |
| Regularization $\lambda$ | ${10}^{-3}$ | Suppresses excessive transition growth |
| Residual saturation $c_{\mathrm{scale}}$ | 0.8 | Controls learning-rate saturation |
| Neighbor-radius factor $c_{r}$ | 1.75 | Radius relative to median nearest-neighbor spacing |
| Spatial-decay factor $c_{\sigma}$ | 1.0 | Gaussian scale relative to median nearest-neighbor spacing |
| Minimum valid neighbors | 1 | Minimum number required for neighborhood fusion |
| Transition-fusion strength $\gamma_{A}$ | 0.6 | Blends local and neighborhood transition matrices |

## S2.10. Computational complexity and memory requirements

The RDAKF signal-estimation stage and the PDE-constrained QPAT reconstruction stage are executed sequentially rather than jointly solved as a single optimization problem. After each sparse-pattern acquisition, RDAKF first estimates a spatially complete set of differential PA waveforms. These estimated waveforms are subsequently used as the input to the iterative QPAT reconstruction. Accordingly, the computational requirements of the two stages are analyzed separately.

For the RDAKF stage, let $M$ denote the waveform-state dimension, $L$ the total number of candidate scanning locations, $S$ the number of directly sampled locations in the current sparse pattern, and $E$ the number of active spatial-neighborhood links. One RDAKF update refers to the complete processing performed after the acquisition of one sparse pattern.

At every candidate location, the state-error covariance is propagated using full $M\times M$ matrices, resulting in a cost of $O\left( LM^{3} \right)$. At the $S$ sampled locations, the full-matrix Kalman measurement correction introduces an additional cost of $O\left( SM^{3} \right)$, whereas the residual-driven transition-matrix gradient and state-vector operations require $O\left( SM^{2} \right)$. The distance-weighted fusion of neighboring $M\times M$ transition matrices requires $O\left( EM^{2} \right)$. Therefore, the computational complexity of one sparse-pattern RDAKF update is $O\left( \left( L+S \right)M^{3}+SM^{2}+EM^{2} \right).$

Because $S\leq L$, the leading-order complexity may be summarized as $O\left( LM^{3}+EM^{2} \right).$ The cubic term is dominated by the full-matrix covariance propagation and Kalman correction. Although the observation matrix is fixed as $\mathbf{H}=\mathbf{I}_{M}$ in the reported implementation, evaluation of the full innovation covariance and Kalman gain still involves dense matrix operations and linear-system solution. The dominant persistent-memory requirement is $O\left( LM^{2} \right).$

This memory requirement arises because each location independently retains full transition and state-error covariance matrices, together with the associated noise-covariance information. These expressions apply to the full-matrix implementation used in this study; no diagonal, sparse, structured, or reduced-rank approximation was employed.

For the optical component of the QPAT reconstruction, let $N_{e}$ denote the number of finite elements, $\mathrm{nnz}\left( \mathbf{K} \right)$ the number of nonzero entries in the optical system matrix, $n_{\mathrm{PCG}}$ the number of preconditioned conjugate-gradient iterations, and $N_{\mathrm{rhs}}$ the number of optical right-hand sides. The optical diffusion equation is discretized using sparse finite elements and solved using incomplete-Cholesky-preconditioned conjugate gradients. Its computational cost is governed by finite-element matrix assembly, incomplete-Cholesky preconditioner construction, and sparse matrix-vector operations during the PCG iterations. The leading-order optical cost can therefore be expressed as $O\left[ N_{e}+C_{\mathrm{ichol}}+N_{\mathrm{rhs}}n_{\mathrm{PCG}}\left( \mathrm{nnz}\left( \mathbf{K} \right)+nnz\left( \mathbf{L}_{\mathrm{ichol}} \right) \right) \right],$ where $C_{\mathrm{ichol}}$ denotes the cost of constructing the incomplete-Cholesky factor $\mathbf{L}_{\mathrm{ichol}}$. Constant factors associated with the optical forward and adjoint solves are omitted from the asymptotic notation.

The acoustic forward and adjoint calculations are implemented using three-dimensional k-Wave propagation. Let $M_{a}$ denote the number of points in the effective acoustic grid and $N_{t}$ the number of propagation time steps. Because the pseudospectral wave propagation requires multidimensional fast Fourier transforms at each time step, the leading computational complexity of one acoustic forward or adjoint propagation is approximately $O\left( N_{t}M_{a}\log M_{a} \right).$

Each QPAT iteration contains both acoustic forward and adjoint propagations, together with the corresponding optical forward and adjoint calculations. Omitting constant factors, the overall leading complexity of one QPAT iteration can therefore be summarized as $O\left[ N_{e}+C_{\mathrm{ichol}}+N_{\mathrm{rhs}}n_{\mathrm{PCG}}\left( \mathrm{nnz}\left( \mathbf{K} \right)+nnz\left( \mathbf{L}_{\mathrm{ichol}} \right) \right)+N_{t}M_{a}\log M_{a} \right].$

Because asymptotic complexity alone does not fully describe practical latency, the measured execution times and memory requirements of the RDAKF and QPAT stages are reported separately in Table S5. The full-matrix RDAKF implementation described above was profiled for a representative acquisition-step update under *S* = 1, 3, and 6 using identical waveform dimensions and parameter settings. The elapsed time denotes the wall-clock time required to complete one recursive full-field waveform-estimation update after acquisition of a sparse pattern. The reported RDAKF memory corresponds to the aggregate footprint of the selected key temporary variables recorded during the profiled update and does not include all persistent location-specific transition, covariance, and state matrices retained by the algorithm.

As summarized in Table S5, the benchmark used data from 36 spatial detection locations, with each PA waveform comprising $M = 281 data points$. Increasing $S$ from 1 to 6 increased the measured RDAKF update time per acquisition step from 25.69 to 42.60 ms, owing to the additional measurement-correction operations at the increased number of sampled locations. In contrast, the aggregate memory footprint of the monitored principal temporary variables increased only modestly, from 136.51 to 142.53 MB. At fixed $L$ and $M$, the dominant persistent storage of the full-matrix implementation is primarily determined by the location-specific transition and covariance matrices and is therefore largely independent of the number of locations sampled within an individual pattern. The reported RDAKF time represents computation-only throughput for one recursive waveform-estimation update after a sparse pattern has already been acquired. It excludes mechanical scanning, laser firing and signal acquisition, data transfer, preprocessing, and all subsequent QPAT reconstruction computations. It therefore does not represent the end-to-end imaging rate or achievable experimental temporal resolution. For the QPAT stage, the optical forward, acoustic forward, and adjoint operations required 1.6067, 1.8946, and 2.4923 s per iteration, respectively. Their combined execution time was approximately 5.9936 s per iteration. These measurements characterize the present MATLAB implementation and profiling hardware and should not be interpreted as hardware-independent complexity bounds.

Table S5. Representative computation times and key temporary-memory footprints of the RDAKF signal-recovery and QPAT reconstruction modules.

| **Computational module** | **Configuration or operation** | **Elapsed time** | **Key temporary memory** |
| --- | --- | --- | --- |
| RDAKF signal recovery | *S* = 1 | 25.69 ms/RDAKF update | 136.51 MB |
|  | *S* = 3 | 30.88 ms/RDAKF update | 138.92 MB |
|  | *S* = 6 | 42.60 ms/RDAKF update | 142.53 MB |
| QPAT reconstruction | Optical forward solution | 1.6067 s/iteration | 7150.35 MB |
|  | Acoustic forward solution | 1.8946 s/iteration |  |
|  | Adjoint solution | 2.4923 s/iteration |  |

**Notes:** All computations were performed in MATLAB R2025b on a workstation running Windows 11 Pro and equipped with a 13th Gen Intel Core i7-13700 CPU, 31.75 GB of RAM, and an NVIDIA GeForce RTX 4060 Ti GPU with 16 GB of memory.

## S2.11. Algorithmic parameters and implementation settings

The principal algorithmic parameters used for RDAKF-based signal estimation and differential QPAT reconstruction are summarized in Table S6. A node-wise nonnegativity projection was applied after each QPAT reconstruction update in all reported simulations, phantom experiments, ex vivo experiments, and comparison reconstructions.

Table S6. Principal parameters used for RDAKF signal estimation and differential QPAT reconstruction.

| **Module** | **Parameter** | **Symbol** | **Simulation** | **Phantom/Ex vivo experiment** |
| --- | --- | --- | --- | --- |
| RDAKF | Transition-model learning rate | $\eta₀$ | 0.06 | 0.06 |
| RDAKF | Initial transition coefficient | $a₀$ | 1 | 1 |
| RDAKF | Weighting factor | $\gamma_{A}$ | 0.6 | 0.6 |
| RDAKF | Initial posterior covariance scale | $P₀$ | 10 | 10 |
| RDAKF | Initial process-noise covariance scale | $Q₀$ | 0.011 | 0.023 |
| RDAKF | Initial measurement-noise covariance scale | $R₀$ | 1 at 1% noise; 10 at 3.16% noise | 1 |
| QPAT | Inter-frame warm-start factor | $\beta_{ws}$ | 0.6 | 0.6 |
| QPAT | Reconstruction step size | $\sigma$ | 0.02 | 0.02 |
| QPAT | Maximum iterations per frame | $N_{max}^{it}$ | 30 | 30 |
| QPAT | Stopping threshold | $\varepsilon_{stop}$ | 2.0 × 10⁻³ per iteration | 2.0 × 10⁻³ per iteration |
| QPAT | Node-wise nonnegativity projection | Δμa,k ≥ 0 | Applied after each update | Applied after each update |

# Supplementary Note S3. Additional evaluation of dynamic QPAT under the limiting sparse-sampling condition

## S3.1. Quantitative reconstruction under increased noise

Fig. S8 presents the corresponding quantitative evaluation at the higher noise level of *η*_noise_ = 3.16% under the extreme sampling condition of *S* = 1. Compared with the results at *η*_noise_ = 1% reported in the main text, the increased noise generally amplified fluctuations in the reconstructed temporal profiles and reduced the structural and contrast-related metrics. The deterioration was most pronounced for Sparse-TTV, which directly performed quantitative reconstruction from the sparse differential photoacoustic observations without full-field signal recovery.

Fig. S8. Quantitative comparison of dynamic QPAT reconstruction obtained with five sparse-data processing strategies under the limiting sampling condition of *S* = 1 and a noise level of *η*_noise_ = 3.16%. (a–c) Target-specific results for Targets 1–3. The left column shows the temporal profiles of the mean reconstructed absorption-coefficient perturbation, mean ${\Delta\mu}_{a}$; the middle and right columns show target-wise SSIM and CNR, respectively. Black dashed curves denote the ground truth, and the colored curves denote Sparse-TTV, linear interpolation, fixed state-transition matrix (fixed-**A** KF), RDAKF without weight fusion (RDAKF w/o WF), and the complete RDAKF. Shaded bands indicate mean ± SD. (d) Target-wise PCC, RMSE, and absolute temporal delay for the five methods.

As shown by the mean ${\Delta\mu}_{a}$ curves in Fig. S8(a–c), Sparse-TTV exhibited substantial amplitude biases for all three targets, including marked late-stage overestimation for Target 1 and progressively increasing positive deviations for Targets 2 and 3. Linear interpolation and the fixed-**A** Kalman filter reduced these errors, but residual amplitude distortion, temporal smoothing, and delayed responses remained, particularly around rapid local changes. RDAKF w/o WF improved temporal tracking through residual-driven adaptation of the state-transition dynamics, although local fluctuations and overshoots were still visible at several acquisition steps. In comparison, the complete RDAKF followed the ground-truth temporal evolution more consistently across the three targets and substantially limited the noise-induced amplitude drift.

The target-wise SSIM and CNR results further demonstrate the benefit of full-field signal recovery before quantitative inversion. Sparse-TTV generally produced the lowest and most strongly fluctuating SSIM and CNR values. The four signal-recovery-based strategies improved both metrics, whereas the complete RDAKF maintained high or near-high SSIM and CNR values over most of the dynamic interval. Although the best-performing method at individual acquisition steps varied among the full-field recovery strategies, neighborhood-weighted fusion generally stabilized the reconstructed target structures and contrast by suppressing noise-driven local prediction errors and their propagation into the PDE-constrained inversion.

## S3.2. Reconstruction of heterogeneous sinusoidal absorption dynamics

To further validate the ability of RDAKF to reconstruct heterogeneous temporal dynamics under highly sparse spatial sampling, a simulation containing 36 spatial sampling locations was performed, with only one location acquired at each sparse acquisition step ($S=1$). Over the total monitoring duration of 1440 s, the absorption coefficients of Target 1, Target 2, and Target 3 followed independent sinusoidal variations comprising 2, 3, and 4 complete cycles, corresponding to periods of 720, 480, and 360 s, respectively. This configuration enabled the temporal reconstruction performance to be evaluated under progressively faster dynamic variations while maintaining identical spatial sampling conditions.

Fig. S9 shows representative ground-truth and RDAKF-reconstructed distributions of the absorption coefficient change, $\Delta\mu_{a}$, at $T_{k}= 600$, $900$, and $1400$ s. Because the three targets had different oscillation periods, they occupied different phases of their respective sinusoidal trajectories at the same observation time. The reconstructed images reproduced these instantaneous contrast differences and correctly localized the three target regions. Although moderate spatial spreading and low-level background artifacts were present, the principal target locations and their relative absorption states remained distinguishable throughout the monitoring interval.

The target-wise temporal results are presented in Fig. S10. For all three targets, the reconstructed mean $\Delta\mu_{a}$ curves followed the corresponding ground-truth trajectories and retained the prescribed 2, 3, and 4-cycle oscillatory patterns. The principal peaks and troughs were consistently recovered, demonstrating that RDAKF preserved the dominant periodic characteristics despite the highly sparse acquisition setting.

The SSIM and CNR curves exhibited periodic variations associated with the instantaneous target amplitudes. Both metrics generally decreased when a target approached the trough of its sinusoidal trajectory, where the target-to-background contrast and target observability were reduced. Therefore, the periodic reductions in SSIM and CNR reflect not only reconstruction errors but also the intrinsically weaker spatial contrast at low absorption amplitudes.

Fig. S9. Representative spatial reconstructions of three absorbers undergoing sinusoidal absorption variations with different temporal periods. Ground-truth and RDAKF-reconstructed distributions of the absorption coefficient change, $\Delta\mu_{a}$, are shown at $T_{k}=600$, 900, and 1400 s. Over the total monitoring duration of 1440 s, Target 1, Target 2, and Target 3 completed 2, 3, and 4 sinusoidal cycles, corresponding to temporal periods of 720, 480, and 360 s, respectively. The left and right columns show the ground-truth and RDAKF-reconstructed results, respectively. Cyan dashed circles indicate the true spatial boundaries of the targets. All images are displayed using a common color scale.

As the temporal frequency increased from Target 1 to Target 3, PCC gradually decreased and RMSE increased, indicating a moderate reduction in temporal reconstruction accuracy for faster dynamics. This trend can be attributed to the larger signal variation between consecutive sparse observations at shorter periods, which increases sensitivity to state-propagation errors, model mismatch, and measurement noise. Nevertheless, the primary oscillatory behavior of all three targets remained well preserved, and no substantial loss of the prescribed periodic patterns was observed.

Fig. S10. Period-dependent quantitative evaluation of RDAKF for three sinusoidally varying absorbers. Over the 1440-s monitoring interval, Target 1, Target 2, and Target 3 completed 2, 3, and 4 sinusoidal absorption cycles, corresponding to temporal periods of 720, 480, and 360 s, respectively. Rows (a–c) show the target-wise mean absorption coefficient change, $\Delta\mu_{a}$, structural similarity index (SSIM), and contrast-to-noise ratio (CNR) as functions of the sparse-pattern acquisition completion time. In the mean $\Delta\mu_{a}$ plots, the black dashed curves denote the ground truth, the blue solid curves denote the RDAKF reconstructions, and the shaded regions represent the spatial mean ± SD within the corresponding target regions. (d) Summary metrics for the three targets, including the Pearson correlation coefficient (PCC), root-mean-square error (RMSE), and absolute temporal delay.

The absolute temporal delays for Target 1, Target 2, and Target 3 were approximately 30, 25, and 30 s, respectively. These values remained at comparable levels and did not exhibit a monotonic increase with temporal frequency. Overall, the results in Figs. S9 and S10 demonstrate that RDAKF can recover the spatial locations and distinct periodic absorption dynamics of multiple targets under the challenging condition of $S = 1$, although the temporal reconstruction accuracy gradually decreases as the underlying dynamics become faster.

# Supplementary Note S4. Independent calibration of the dynamic absorption profile

## S4.1. Transmission photon-counting calibration system and solution preparation

To independently evaluate the feasibility of the flow-mixing model for predicting temporal absorption changes, a separate transmission photon-counting experiment was performed to assess the agreement between the measured and model-predicted temporal trends.

Two stock solutions, solution A($S_{A}$)and solution B($S_{B}$), were used to generate controlled absorption changes. At 1064 nm, $S_{A}$ and $S_{B}$ had absorption coefficients of 0.200 and 0.0244 ${mm}^{-1}$, respectively. The continuously stirred mixing reservoir initially contained 60 mL of solution C ($S_{C}$), with an absorption coefficient of $0.0144\mathrm{mm}^{-1}$. Depending on the experimental stage, either $S_{A}$ or $S_{B}$ was delivered into the reservoir, while the mixed solution was withdrawn and transported to the measurement channel.

The mixed solution flowed through a 2 × 2 mm² square channel and was transversely illuminated by a 1064-nm laser diode (U-LD-102071D, Union Optronics Corp., Taiwan). Transmitted light was collected by a lens and detected using an InGaAs/InP SPAD module (ID Qube NIR, ID Quantique, Switzerland). Photon counts were recorded by an SPC-130 module (Becker & Hickl GmbH, Germany) with a 0.5-s integration time. Fluid delivery and withdrawal were controlled by two independently operated peristaltic pumps (BT100-02, Baoding Qili Constant Flow Pump Co., Ltd., Baoding, China) (Fig. S11).

Fig. S11. Experimental setup for independent photon-counting calibration of the dynamic absorption profile. Solution A or B was delivered into a continuously stirred reservoir by the inlet pump, while the outlet pump transported the mixture through a 2 × 2 mm² square flow channel. Transmitted 1064-nm light was collected and detected by an InGaAs/InP SPAD, with photon counts integrated for 0.5 s.

## S4.2. Absorption-coefficient calculation

The air-reference photon count was acquired using the same channel and optical alignment with the channel filled with air. Dark counts were recorded using the same detection settings with the illumination blocked. Following the Beer-Lambert law, the absorption coefficient was calculated after dark-count correction as

|  | $\text{μ}_{\text{a}}\left( \text{t} \right)=\frac{\text{1}}{\text{L}}\ln\left[ \frac{\text{I}_{\text{air}}\text{−}\text{I}_{\text{dark}}}{\text{I}\left( \text{t} \right)\text{−}\text{I}_{\text{dark}}} \right],$ | (S32) |
| --- | --- | --- |

where $\text{I}\left( \text{t} \right)$ is the photon count transmitted through the liquid sample at time $t$, $\text{I}_{\text{air}}$ is the reference photon count obtained with the same channel filled with air, $\text{ }\text{I}_{\text{dark}}$ is the dark photon count recorded with the illumination blocked, and $\text{L}$ = 2 mm is the optical path length through the liquid.

## S4.3. Dynamic protocols and flow-mixing model

The five-stage calibration comprised five consecutive 600-s conditions: (1) $S_{A}$ infusion with inlet/outlet pump settings of 15/15; (2) $S_{B}$ infusion at 15/15; (3) $S_{A}$ infusion at 30/10, for which inflow exceeded outflow; (4) $S_{B}$ infusion at 10/30, for which outflow exceeded inflow; and (5) cessation of both pumps at 0/0. Pump settings were converted to flow rates using the calibrated coefficient of 0.7666 mL/min per setting unit.

Two additional 600-s protocols were used to evaluate rapid absorption changes. During continuous infusion of solution $S_{A}$, a volume of solution $S_{B}$ equal to the liquid volume in reservoir C was rapidly added to the reservoir at approximately 300 s on the original experimental protocol clock. In the second protocol, reservoir C was initially filled with $S_{A}$, solution $S_{B}$ was infused continuously, and an equal reservoir volume of $S_{A}$ was rapidly added at approximately 300 s. Pumping continued after each addition. Six independent runs were performed for each protocol. Because solution addition, mixing, and transport were not instantaneous, small run-to-run variations in the downstream transition timing were expected.

The mixing reservoir was modeled as a perfectly mixed compartment. Within the concentration range used here, the absorption coefficient was assumed to vary linearly with the concentration of the absorbing component. Conservation of liquid volume and absorbing material therefore gives

|  | $\frac{\text{dV}\left( \text{t} \right)}{\text{dt}}=\text{Q}_{\text{in}}-\text{Q}_{\text{out}},\quad\quad\frac{\text{d}\text{μ}_{\text{a}}\left( \text{t} \right)}{\text{dt}}=\frac{\text{Q}_{\text{in}}}{\text{V}\left( \text{t} \right)}\left[ \text{μ}_{\text{a}\text{,in}}\text{−}\text{μ}_{\text{a}}\left( \text{t} \right) \right],$ | (S33) |
| --- | --- | --- |

where $V(t)$ is the liquid volume in reservoir $C$, $\text{Q}_{\text{in}}$ and $\text{Q}_{\mathrm{out}}$ are the inlet and outlet volumetric flow rates, respectively, $\text{μ}_{\text{a}}\left( \text{t} \right)$ is the absorption coefficient of the liquid in reservoir $C$, and $\text{μ}_{\text{a}\text{,in}}$ is the absorption coefficient of the infused solution.

For an abrupt addition, the absorption coefficient immediately after mixing was calculated by volume-weighted conservation:

|  | $\mu_{a,\mathrm{post}}=\frac{V_{\mathrm{pre}}\mu_{a,\mathrm{pre}}+V_{\mathrm{add}}\mu_{a,\mathrm{add}}}{V_{\mathrm{pre}}+V_{\mathrm{add}}},$ | (S34) |
| --- | --- | --- |

where $V_{\mathrm{pre}}$ and $\mu_{a,\mathrm{pre}}$ are the reservoir volume and absorption coefficient immediately before addition, respectively; $V_{\mathrm{add}}$ and $\mu_{a,\mathrm{add}}$ are the volume and absorption coefficient of the added solution; and $\mu_{a,\mathrm{post}}$ is the absorption coefficient immediately after homogeneous mixing. For the equal-volume perturbations used here, $V_{\mathrm{add}}$=$V_{\mathrm{pre}}$, and the expression reduces to

|  | $\mu_{a,\mathrm{post}}=\frac{\mu_{a,\mathrm{pre}}+\mu_{a,\mathrm{add}}}{2},$ | (S35) |
| --- | --- | --- |

## S4.4. Transport-delay measurement and experimental validation

Because the flow-mixing model describes the absorption-coefficient evolution in reservoir C, whereas the photon-counting measurements were acquired in a downstream flow channel, a temporal delay was expected owing to fluid transport through the connecting tubing. The reservoir-to-channel transport delay was determined by manually timing the fluid transit and was approximately 60 s. Accordingly, for the abrupt-perturbation protocols, the perturbation introduced at approximately 300 s on the experimental protocol clock reached the downstream measurement channel at approximately 360 s. This fixed delay was incorporated into the model predictions for all runs and dynamic protocols, while the measured trajectories retained their original experimental time coordinates.

The curves in Figs. S12 and S13 are therefore presented on the original experimental protocol clock, with the measured transport delay incorporated into the corresponding model predictions. Measurements are reported as the mean ± SD of six independent runs, and the RMSE values were calculated between the measured mean and the delayed model prediction. For the five-stage experiment, the measured trajectory agreed with the model prediction with an overall RMSE of 0.0082 mm^−1^ (Fig. S12). The remaining discrepancies were most evident during system startup and near pump-switching or abrupt-perturbation transitions.

For the $S_{A}$-infusion/$S_{B}$-perturbation and $S_{B}$-infusion/$S_{A}$-perturbation experiments, the overall RMSE values were 0.0084 and 0.0069 mm−1, respectively (Fig. S13). The larger localized errors near the downstream perturbation arrivals at approximately 360 s can be attributed to finite solution-addition time, transient concentration heterogeneity, pump response, incomplete mixing, temporal integration, and transport dispersion. Away from these transitions, the measured profiles reproduced the predicted direction, magnitude evolution, and recovery behavior.

Fig. S12. Experimental validation of the five-stage dynamic absorption profile. (a) Measured absorption dynamics and the flow-mixing model for five consecutive conditions: A infusion (15/15), B infusion (15/15), A infusion with inflow exceeding outflow (30/10), B infusion with outflow exceeding inflow (10/30), and pumps off (0/0). The solid line and shaded band indicate the mean and SD of six independent runs, thin lines show individual runs, and the dashed line denotes the model. (b) Residual e(t), defined as measured mean minus model; the shaded band represents residual ± SD.

Fig. S13. Experimental validation of rapid absorption changes produced by abrupt perturbations. (a) Continuous infusion of solution $S_{A}$ with an equal-volume addition of solution $S_{B}$, and (b) continuous infusion of solution $S_{B}$ with an equal-volume addition of solution $S_{A}$. (c, d) Corresponding residuals between the measured mean and the model prediction. The perturbation solutions were added to reservoir C at approximately 300 s on the original experimental protocol clock. Owing to the manually measured reservoir-to-channel transport delay of approximately 60 s, the corresponding perturbations appeared in the downstream measurement channel at approximately 360 s, as indicated by the vertical dotted lines. Small run-to-run variations in the observed transition timing arose from finite addition, mixing, pump response, and fluid transport. Solid blue lines and shaded bands indicate the mean and SD of six independent runs, thin blue lines show the individual runs, and dashed black lines denote the flow-mixing model incorporating the measured transport delay.
